# Dirac gap opening and Dirac-fermion-mediated magnetic coupling in antiferromagnetic Gd-doped topological insulators and their manipulation by synchrotron radiation

**DOI:** 10.1038/s41598-019-41137-w

**Published:** 2019-03-18

**Authors:** A. M. Shikin, D. A. Estyunin, Yu. I. Surnin, A. V. Koroleva, E. V. Shevchenko, K. A. Kokh, O. E. Tereshchenko, S. Kumar, E. F. Schwier, K. Shimada, T. Yoshikawa, Y. Saitoh, Y. Takeda, A. Kimura

**Affiliations:** 1 Saint Petersburg State University, Saint Petersburg, 198504 Russia; 2 Novosibirsk State University, Novosibirsk, 630090 Russia; 3 V.S. Sobolev Institute of Geology and Mineralogy, Novosibirsk, 630090 Russia; 4 A.V. Rzhanov Institute of Semiconductor Physics, Novosibirsk, 630090 Russia; 5 Hiroshima Synchrotron Radiation Center, Hiroshima University, Hiroshima, 739-0046 Japan; 6 Materials Sciences Research Center, Japan Atomic Energy Agency, Hyogo, 679-5148 Japan; 7 Department of Physical Sciences, Graduate School of Science, Hiroshima University, Hiroshima, 739-8526 Japan

## Abstract

A new kind of magnetically-doped antiferromagnetic (AFM) topological insulators (TIs) with stoichiometry Bi_1.09_Gd_0.06_Sb_0.85_Te_3_ has been studied by angle-resolved photoemission spectroscopy (ARPES), superconducting magnetometry (SQUID) and X-ray magnetic circular dichroism (XMCD) with analysis of its electronic structure and surface-derived magnetic properties at different temperatures. This TI is characterized by the location of the Dirac gap at the Fermi level (E_F_) and a bulk AFM coupling below the Neel temperature (4–8 K). At temperatures higher than the bulk AFM/PM transition, a surface magnetic layer is proposed to develop, where the coupling between the magnetic moments located at magnetic impurities (Gd) is mediated by the Topological Surface State (TSS) via surface Dirac-fermion-mediated magnetic coupling. This hypothesis is supported by a gap opening at the Dirac point (DP) indicated by the surface-sensitive ARPES, a weak hysteresis loop measured by SQUID at temperatures between 30 and 100 K, XMCD measurements demonstrating a surface magnetic moment at 70 K and a temperature dependence of the electrical resistance exhibiting a mid-gap semiconducting behavior up to temperatures of 100–130 K, which correlates with the temperature dependence of the surface magnetization and confirms the conclusion that only TSS are located at the E_F_. The increase of the TSS’s spectral weight during resonant ARPES at a photon energy corresponding to the Gd 4*d*-4*f* edge support the hypothesis of a magnetic coupling between the Gd ions via the TSS and corresponding magnetic moment transfer at elevated temperatures. Finally, the observed out-of-plane and in-plane magnetization induced by synchrotron radiation (SR) due to non-equal depopulation of the TSS with opposite momentum, as seen through change in the Dirac gap value and the *k*_∥_-shift of the Dirac cone (DC) states, can be an indicator of the modification of the surface magnetic coupling mediated by the TSS.

## Introduction

In recent years, magnetically-doped TIs attracted great interest due to the possible realization of the Quantum Anomalous Hall effect (QAHE)^1–7^ and topological magneto-electric effect^6,7^ caused by magnetic impurities with a high out-of-plane magnetic anisotropy^8–12^. Realizing these effects, the problems of the 2D surface ferromagnetism and the Dirac-fermion-mediated surface coupling are holding a special place^12–15^. The out-of-plane magnetization makes the surface Dirac fermions massive^8,9^ resulting in a gap opening at the DP^8,9,11,13^ and formation of chiral edge states allowing the realization of the QAHE^5,16,17^. The highest QAHE is observed, when the carrier density decreases to a such low level, that the 1D chiral edge states are not disturbed^5,16–19^. Therefore, for the effective realization of the QAHE in magnetically-doped TIs the Dirac gap must be located at the E_F_^3–5,16–19^, and the developed surface magnetic coupling must survive in the insulating regime, i.e. should be independent form bulk carriers. As for the DP location, it was recently shown^5,13,17^ that the magnetic field dependence of the Hall resistance, measured for Cr_0.22_(Bi_*x*_Sb_1−*x*_)_1.78_Te_3_ at different concentrations of Sb, has a pronounced maximum under transition from p- to n-type, i.e. for the compounds with the Dirac gap localization at the E_F_^5,16,17^. A similar situation takes place for the most effective charge-to-spin current transformation^20^ and under variation of applied gate voltage, when it shifts the DP (and corresponding gap) close to the E_F_ position^5,16,17^. At the same time, a character of magnetic coupling providing the QAHE is very important. Recently, Kou *et al*.^18,19^ who studied the mechanism of ferromagnetic (FM) ordering and its connection with the DP position relative to the E_F_, showed that a shift of the Dirac gap towards E_F_ is followed via the transition from a hole-mediated RKKY coupling to an electric-field-independent bulk van Vleck ferromagnetism. It means that classical RKKY-type FM coupling through itinerant carriers cannot be directly applied for analysis of the conditions of the QAHE realization and cannot be used for detailed analysis of the developed surface magnetism when the Dirac gap is located at the E_F_.

The QAHE assumes a dissipationless transport occurring via chiral edge channels located inside the magnetic gap^16^, which is caused by the exchange coupling between the surface states and the FM impurity magnetic moments. However, at finite temperatures the 1D non-chiral edge channels can be added due the electrons excited from the valence and conduction band (VB and CB) states^18,20^. Therefore, for effective realization of the chiral 1D edge transport (and, consequently, of the QAHE) the Dirac gap should be located at the E_F_ without the VB and CB states crossing the E_F_. In such a case, a 2D surface Dirac-fermion-mediated ferromagnetism (i.e. surface ferromagnetism mediated by the DC topological surface states) should be significantly enhanced.

Such surface Dirac-fermion-mediated ferromagnetism (discussed in refs^12,13^) can provide a magnetic ordering or coupling at elevated temperatures due to its different nature and can have a stronger effect on the QAHE^14,15,21^. The surface Dirac-fermion-mediated magnetic coupling arises as a result of the interaction between the local spins of magnetic impurities and electrons in the surface DC states and, therefore, can develop without the necessity for bulk ferromagnetism. Thereat, the surface Curie temperature for magnetically doped TIs is assumed to be higher than that for the bulk^21^. The surface magnetic coupling can then be seen to force the opening of a gap at the DP in magnetically-doped TIs^8,9,11,13,22^ at temperatures higher than the bulk magnetic transition.

Recently, the possibility of 2D (or flat) magnetism at room temperature and its manipulation via the application of an electric field sourced from to the magnetoelectric effect were shown^23–25^ which were also strongly related to the interplay between low-dimensional magnetic and topological properties. Moreover, for the bilayer system EuS/Bi_2_Te_3_ a possibility of topologically enhanced interface magnetism was observed^26,27^ due to coupling of a ferromagnetic insulator (EuS) to the topological insulator (Bi_2_Te_3_). It was shown that strong enhancement of the exchange interaction among Eu atoms is due to the additional coupling channel via the TSS at the interface. It was observed that the developed magnetism can persist up to room temperature, despite the fact that the Curie temperature of bulk EuS is only 17 K. Owing to the short-range nature of the FM exchange interaction, Time-Reversal Symmetry (TRS) is broken only near the surface of the TI, while leaving its bulk states unaffected.

The current work is devoted to comparative study of electronic structure and magnetic properties of a new class of magnetic rare-earth (RE) - doped AFM TIs with stoichiometry Bi_1.09_Gd_0.06_Sb_0.85_Te_3_ (in comparison with more classical one Bi_1.09_V_0.06_Sb_0.85_Te_3_). These compounds are both characterized by localization of the Dirac gap at the E_F_ without partial occupation of either CB or VB states present at the surface, a condition important for the effective realization of QAHE. It is known that for pristine TI with stoichiometry (Bi_1−*x*_Sb_*x*_)_2_Te_3_^5,17,28^ the DP is arranged at the Fermi level, when the Sb-concentration lies in the region between 0.88–0.94. If the E_F_ traverses the DP, the 2D resistance reaches the maximum and shows insulating behavior which reflects the depletion of electron-type bulk carriers. The key challenge for realization of the QAHE in the magnetically-doped TIs with the stoichiometry mentioned above is to achieve simultaneously a low bulk carrier density together with an appropriate ferromagnetic ordering. Unfortunately, the incorporated TM atoms act not only as magnetic dopants. The doping by transition metal (TM) magnetic impurities leading to the Dirac gap opening is followed also by the charge carrier doping effect. TMs are usually divalent and, therefore, substitution of Bi^3+^ with TM^2+^ leads to additional hole doping. Therefore, it is hard to distinguish the effect related only to the 1D edge states and corresponding magnetic ordering. On the other hand, if the doping elements are rare-earth metals such as Gd, which are expected to be at least trivalent, no hole doping will take place and only magnetic moments can be induced through Gd-doping^29^. Gd has an electronic configuration [Xe]4*f* ^7^(5*d* 6*s*)^3^. It has a half-filled *f*-shell with a large magnetic moment arising from the 7 unpaired 4*f*-spins and an equal number of bonding electrons compared to Bi. Under Gd-doping the lattice parameters of the host TI are not changed.

The current work aims to perform a comparative analysis of electronic and surface magnetic properties of the Gd-doped TI with stoichiometry Bi_1.09_Gd_0.06_Sb_0.85_Te_3_ which is characterized by an AFM coupling in the bulk at low temperatures and a Dirac gap located near E_F_. The properties are related to the possibility of a surface Dirac-fermion-mediated magnetic coupling in this compound at temperatures higher than the AFM/PM transition. Such TIs are expected to be useful for the realization of the QAHE and its manipulation. In order to confirm the role of the TSS in the magnetic coupling, we have also investigated changes of the Dirac gap value and a *k*_∥_-shift of the DC states with opposite momentum and spin orientation by linear and circularly polarized SR under non-equal photoexcitation.

## Experiment

### Bi_1.09_Gd_0.06_Sb_0.85_Te_3_

**Figure 1:**
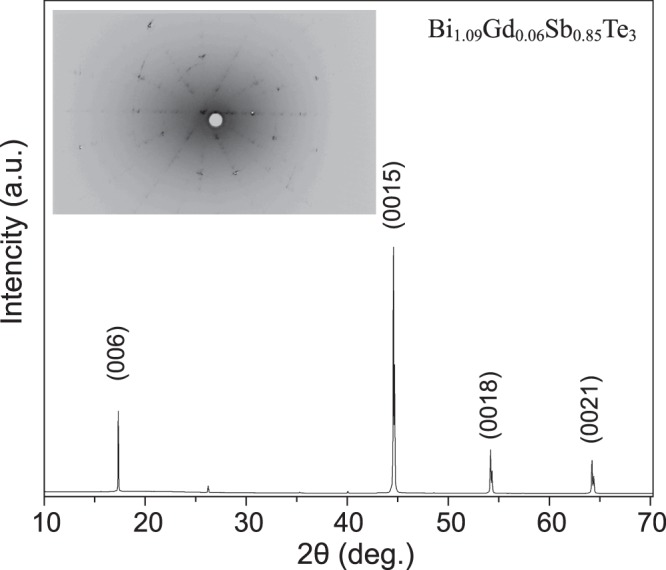
X-ray diffraction pattern of Bi_1.09_Gd_0.06_Sb_0.85_Te_3_ single crystal. Inset shows the Laue diffraction pattern which reveals the high crystalline quality of the sample.

The sample of Bi_1.09_Gd_0.06_Sb_0.85_Te_3_ was characterized by X-ray diffractometer with Cu K_α_ radiation (*λ* = 1.5406 Å) operating at 40 kV and 30 mA. Figure 1 shows the XRD data of Bi_1.09_Gd_0.06_Sb_0.85_Te_3_ single-crystal, where the diffraction peaks corresponding to the (001) family reflections of the rhombohedral crystal structure with space group $R\bar{3}m$ $({D}_{3d}^{5})$, which indicates that the crystal growth direction is along its c-axis. No impurity peaks were found in the XRD which confirms that the sample remains in a single phase. This reveals that Bi_1.09_Gd_0.06_Sb_0.85_Te_3_ crystallizes in the same structure as Bi_2_Te_3_^30,31^. From the XRD the following lattice parameters were extracted *a* = *b* = 4.34(7) Å, *c* = 30.56(1) Å, *V* = 498.49(2) Å^3^ respectively. Inset of Fig. 1 shows the Laue diffraction pattern of a single crystal of Bi_1.09_Gd_0.06_Sb_0.85_Te_3_, which was acquired in transmission scattering geometry.

**Figure 2:**
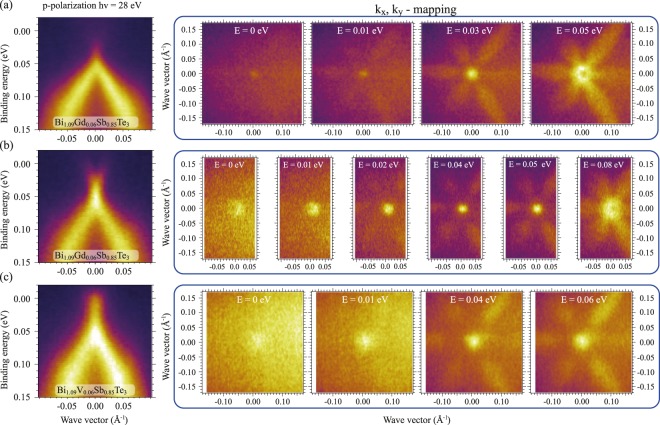
Left column - the ARPES dispersion maps measured for Bi_1.09_Gd_0.06_Sb_0.85_Te_3_ – (**a**,**b**) and for Bi_1.09_V_0.06_Sb_0.85_Te_3_ – (**c**) at temperature 55 K and photon energy of 28 eV. For Bi_1.09_Gd_0.06_Sb_0.85_Te_3_ the ARPES maps with different DP positions relative to the E_F_ are presented (immediately after cleavage of the sample – (**a**) and after long time experiment – (**b**) shifted to higher binding energies). Right columns – series of corresponding *k*_*x*_, *k*_*y*_–mappings of the DC states measured at different binding energies relative to the E_F_. At the binding energies higher 0.03–0.04 eV the “star” -like distribution of the VB states is visible. The ARPES dispersions are measured in the vertical directions of the *k*_*x*_, *k*_*y*_–mappings.

Figure 2a,b (left column of insets) show the ARPES dispersion maps measured for Gd-doped topological insulators with stoichiometry Bi_1.09_Gd_0.06_Sb_0.85_Te_3_ at a temperature of 55 K under photoexcitation by linear p-polarized SR, directly after sample surface cleaving - (a) and after 4–5 hours under residual gas exposure - (b). The dispersions were measured at a photon energy of 28 eV which is characterized by maximal relative intensities of the topological surface states at the DP after corresponding *k*_*x*_, *k*_*y*_–mappings. These mappings are shown as the cuts at different binding energies (E_B_) in right side of Fig. 2a,b. First of all one can see that the DP is really located close to E_F_ for this compound. After long time exposure one can distinguish a small energy shift of the DP towards higher E_B_ of about 0.01–0.02 eV.

**Figure 3:**
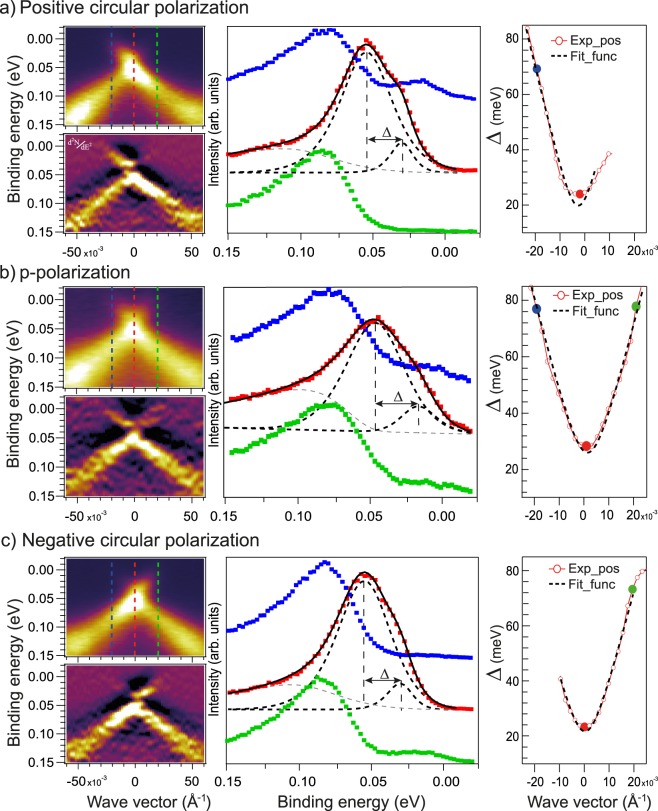
Left column - the ARPES dispersion maps measured for Bi_1.09_Gd_0.06_Sb_0.85_Te_3_ at temperature 55 K and photon energy of 28 eV under photoexcitation by SR of different polarizations (linear p-polarization – (**b**) and circular ones of opposite helicity – (**a**,**c**)). Both the ARPES dispersion maps are presented in the N(E) and *d*^2^*N*/*dE*^2^ forms for better visualization of the electronic structure near the DP. The middle column - the corresponding EDC-profiles measured directly at the Γ-point (*k*_∥_ = 0) and at opposite *k*_∥_ relative to the Fermi level with decomposition on the spectral components (red lines). Right column – the dependence of the estimated splitting between the lower and upper DC states on *k*_∥_.

**Figure 4:**
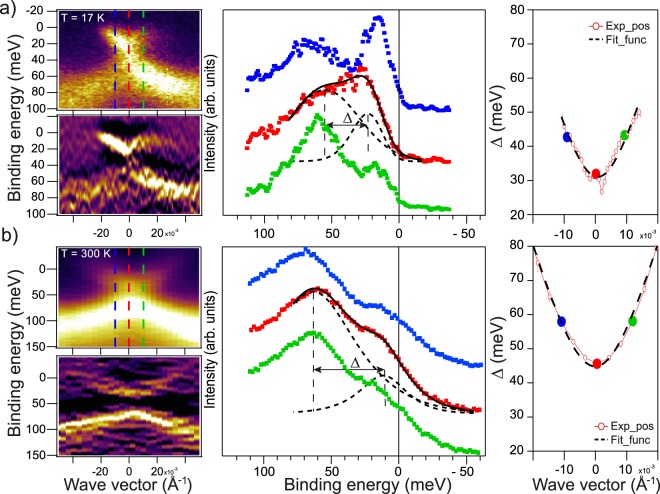
Left column - the ARPES dispersion maps measured for Bi_1.09_Gd_0.06_Sb_0.85_Te_3_ at temperatures 17 K and 300 K at photon energy of 30 eV under photoexcitation by p-polarized SR The ARPES dispersion maps are presented both in the N(E) and *d*^2^*N*/*dE*^2^ forms for better visualization of the electronic structure near the DP. The middle column - the corresponding EDC-profiles measured directly at the Γ-point (*k*_∥_ = 0) and at opposite *k*_∥_ relative to the Fermi level with decomposition on the spectral components (red lines). Right column – the dependence of the estimated splitting between the lower and upper DC states on *k*_∥_.

Figure 3a–c (left columns of insets) demonstrate a comparison between the ARPES dispersion maps measured under photoexcitation by linearly polarized SR (for the same case as presented in Fig. 2b) - (b) and by circularly polarized SR of opposite helicity - (a, c). The spectra were measured at temperature of 55 K. Below each ARPES dispersion maps a second derivatives *d*^2^*N*/*dE*^2^ are shown for better visualization of the electronic structure of the DC states and possible splitting of the DC states at the DP induced by magnetic doping. One can see that the DP and the corresponding DC state splitting are actually located close to the E_F_. For the initial measurements (Fig. 2a) only states of the lower DC are occupied and can be distinguished in the ARPES dispersion maps. Absence of occupied states of the upper DC for this case is clearly seen in the *d*^2^*N*/*dE*^2^ presentation also shown in Supplementary Fig. 1Sa. The structure of the VB states for a wider E_B_ range measured at different photon energies is presented in Supplementary Fig. 2S. After exposed to residual gases and subsequent electron doping, the energy splitting between the upper and lower DCs becomes visible and can be evaluated. These spectra are presented in Fig. 3 and Supplementary Fig. 1Sb. We connect this splitting between the lower and upper DCs to the gap formation at the DP, even though it is not followed by a visible intensity dip at the DP. The latter can be related to a possible heterogeneity of the Gd-impurity distribution and corresponding spatial variation of the Dirac gap value along the surface^17^ or thermal broadening of the ARPES spectral weights. In the middle column in Fig. 3 the Energy Distribution Curves (EDCs) measured directly at the DP (at *k*_∥_ = 0) (red curves) and for opposite momenta relative to the Γ-point (blue and green curves) are shown. For the EDC at the Γ-point the fitting with decomposition into spectral components is shown. The fitting functions are constructed using the line width of the peaks when they are outside of the DP, i.e. when the upper and lower DC states are well separated. The corresponding fitting of the measured EDCs with deconvolution into analogous spectral components was performed at all *k*_∥_-position in the measured ARPES dispersion maps. As a result, the DC state splitting as a function of *k*_∥_-variation Δ(*k*_∥_) along the ARPES dispersion profile were plotted for all used polarization of SR (see red open circles in insets in right column in Fig. 3). Δ(*k*_∥_) from each EDC was then fitted with a model function, which is shown as black dashed lines (see Suppl. inf. section 12). The gap evaluations by standard method of the measured EDC fitting without analysis of the *k*_∥_-dependence of the gap are shown by red, green and blue circles. The results of the gap estimations using all three methods are shown and compared in corresponding insets in right column in Fig. 3 for all used polarization of SR. The final value of the DC splitting at the DP was estimated as the minimal value obtained at the DP using the constructed *k*_∥_-dependences of the DC state splitting. The use of the constructed *k*_∥_-dependences of the DC state splitting allowed us to minimize the errors and arbitrariness in estimating the gap values. As a result, our estimation of the splitting between the lower and upper DC states at the DP measured under photoexcitation by p-polarized SR gives a value of about 26–28 meV. Similar estimations for the cases of photoexcitation by circularly polarized SR of opposite helicity give the values of 21–24 and 23–24 meV, respectively (using different estimation methods). The corresponding uncertainty in the gap value at the DP can be rated as about ±3 meV. To analyze the possible effect of temperature on the splitting of the DC states at the DP Fig. 4 shows similar ARPES dispersion maps measured for the Gd-doped TI with slightly modified stoichiometry Bi_1.19_Gd_0.06_Sb_0.75_Te_3_ at temperatures of 17 K and 300 K, i.e. lower and significantly higher than that used for measurements presented in Fig. 3. For this compound the splitting between the upper and lower DC states is more pronounced. It is interesting that at room temperature the gap at the DP is also observed in the ARPES dispersion maps. The estimations of the Dirac gap (by the method used to analyze the data shown in Fig. 3) give the Dirac gap value of about 30–32 meV (±3–4 meV) at 17 K and of about 45 meV (±5 meV) at 300 K taking into account the peak broadening. The use of a wider angle mode with the higher angle integration for measurements shown in Supplementary Fig. 3S gives the gap value of about 35–36 meV (±5 meV) taking into account the different position on the surface during measurements. Here, we have to note that preservation of the open Dirac gap up to room temperature was also observed for recently discovered new kind of magnetically-ordered TI MnBi_2_Te_4_^32,33^, where the interplay between spin-orbit interaction and magnetic coupling plays a significant role.

As we noted before, Fig. 3 demonstrates the change in the Dirac gap value under photoexcitation by circularly polarized SR of opposite helicity. These changes are related to the out-of-plane and in-plane magnetization generated by SR of different polarization due to non-equivalent photoexcitation of the DC states with opposite momenta (see refs^34,35^ and discussion below) In the presented ARPES dispersion maps one can clearly see the redistribution of the intensity between the DC branches with opposite momentum in upper DC, which is reversed for SR of opposite helicity. The width of the spectral lines in the ARPES dispersion maps under excitation by circularly polarized SR is slightly reduced. Under switching from photoexcitation by linearly-polarized SR to that by circularly polarized SR the value of the Dirac gap (indicated by the splitting between the DC states) reduces from 27 meV (on average) to 22–23 meV (on average).

At the same time, the circular dichroism (CD) signal, which is the result of the subtraction between the ARPES dispersion maps measured at opposite helicity of the SR (see Supplementary Fig. 4S), confirms that TSS characterized by inverse spin orientation are located mainly above the VB states, i.e. above the energy of 0.08–0.09 eV. While for the lower DC states some mixing with the upper edge VB states can be assumed. In Supplementary Fig. 3S the contribution of these states can be separated. The peak of the VB states is located at E_B_ 60 meV in the region of the DP. Thereat, the states of the lower DC expand to E_B_ 40 meV.

We have to note that the ARPES dispersion maps in Figs 3, 4 and Supplementary Fig. 3S and the *k*_*x*_, *k*_*y*_-mapping of the DC states in Fig. 2 show that neither CB nor VB cross E_F_ and therefore the only contribution to a magnetic coupling at the surface is the TSS. The VB states with “star” -like angle distribution are visible in the mappings in Fig. 2 only at E_B_ 0.03–0.04 eV and below. Therefore, we may assume that for Bi_1.09_Gd_0.06_Sb_0.85_Te_3_ with the Dirac gap localization at the E_F_ the contribution of the magnetic coupling mediated by the TSS (the Dirac-fermion-mediated coupling) plays a significant role. Moreover, we may hypothesize that this kind of magnetic coupling could be important at elevated temperatures, when an other long-range magnetic coupling is destroyed and the coupling between the Gd-ions via the TSS may be mediated differently (see discussion below).

**Figure 5:**
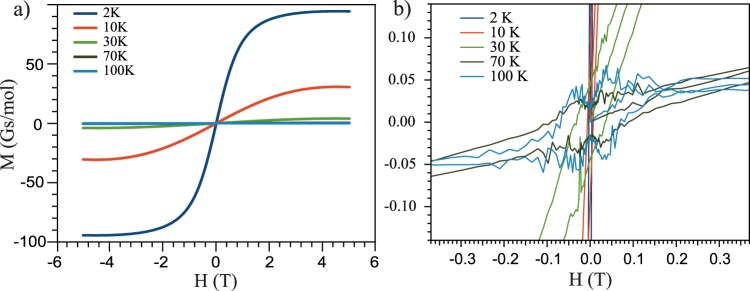
(**a**) The magnetic field dependences of the effective magnetization for Bi_1.09_Gd_0.06_Sb_0.85_Te_3_ measured by SQUID at different temperatures between 2 K and 100 K with external magnetic field applied perpendicular the surface. (**b**) Detailed magnetic field dependences of the effective magnetization presented for lower scale (20 times more sensitive) of the induced magnetization in the region of the low values of applied magnetic field.

To analyze the magnetic properties of Bi_1.09_Gd_0.06_Sb_0.85_Te_3_ we have carried out M(H) and M(T) measurements using a Superconducting Quantum Interference Device (SQUID) magnetometer. Figure 5a shows the dependencies of the magnetization as a function of applied out-of-plane magnetic field (B//c) at different temperatures between 2 K and 300 K. The experimental curves demonstrate the well-known S-like behavior with gradual saturation achieved at 2 T which is typical for PM or AFM^36–41^.This behaviour was reproduced for a second sample (see Fig. 5Sa, for comparison).

**Figure 6:**
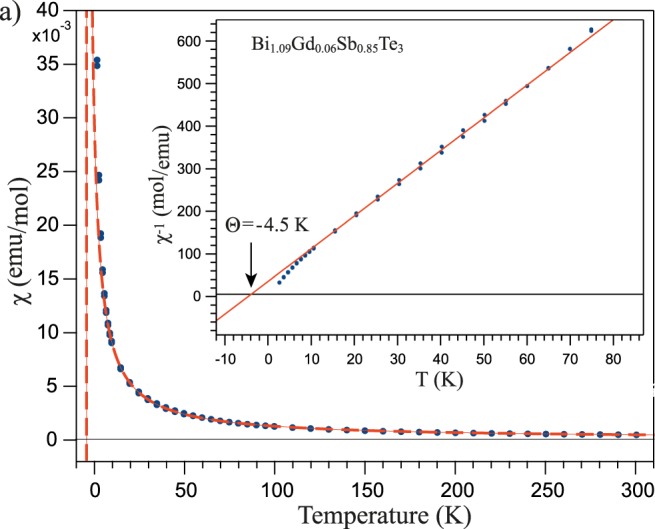
The temperature dependence of the magnetic susceptibility (*χ*) measured for Bi_1.09_Gd_0.06_Sb_0.85_Te_3_ with external magnetic field applied perpendicular the surface with approximation by the Curie-Weiss law at low temperatures (red lines). Insert - the temperature dependence of the inversed magnetic susceptibility 1/(*χ*) with corresponding approximation at low temperatures (red lines) showing the negative values of the Weiss temperature (Θ = −4.5 K).

The temperature dependence of the magnetic susceptibility under applied out-of-plane field of 1 T in the range of temperatures between 2 K and 300 K is presented in Fig. 6. The approximation of the dependence by the Curie-Weiss law (*χ* = *C*/(*T* − Θ), where C is the material-specific Curie constant and Θ is the Weiss temperature) is shown by red dotted line. The temperature dependence 1/(*χ*) is presented as inset in Fig. 6. The fitting and the linear approximation of the 1/(*χ*) dependence clearly demonstrates a negative Weiss temperature (Θ = −4.5 K), which points towards the AFM ordering at low temperatures. Similar measurements for another sample (sample 2) carried out at the applied magnetic field of 5 T gives also a negative Weiss temperature, with a slightly different value (Θ = −8.3 K). Results of these measurements are presented in Supplementary Fig. 6S. Both measurements testify to the bulk AFM coupling at low temperatures that correlates with the results published in the literature for different RE-doped TIs^29,36,38,42^.

At the same time, the effective magnetic moment (*μ*_*eff*_) per Gd atom of 8.1 *μ*B can be estimated from the approximation noted above. Gd has an equal number of bonding electrons to Bi, therefore no free carriers are introduced by the isoelectric substitution of Bi by Gd in Bi_2_Te_3_^36^. This (*μ*_*eff*_) value is close to the theoretical value of 8.0 *μ*B expected for free Gd^3+^ ion^29,36–38,43^. It indicates that Gd substitutes Bi without distortion of the stoichiometry, which would lead to changes of the valency and hence a deviation of the measured magnetic moment^29^. This is further supported by ref.^40^, where it was shown that the Gd^3+^ site in Bi_2(1−*x*)_Gd_2*x*_Te_3_ has *C*_3*ν*_ symmetry, indicating a true substitution of Bi^3+^ by Gd^3+^.

It is important to note that the temperature dependence of the magnetic susceptibility differ significantly from 3*d* substituted Bi_1.31_V_0.03_Sb_0.66_Te_3_ which is characterized by a positive Weiss temperature of Θ = 2.4 K (see Supplementary Fig. 7S) which is representative of traditional TM-doped TIs with a FM ground state. We take this difference in the magnetic susceptibility behavior as an additional evidence of the AFM coupling developed in Bi_1.09_Gd_0.06_Sb_0.85_Te_3_ at low temperatures.

The AFM coupling was also observed for the layered magnetically-doped BiTeI^44,45^, for layered RE-Rh_2_Si_2_ systems (where RE is Gd, Ho)^41^ and for recently discovered magnetically-ordered stoichiometric AFM TI MnBi_2_Te_4_^32,33,46^, where the magnetic coupling is provided by an AFM coupling between the magnetic Mn atoms in the neighbor quintuple layers with alternating magnetic moments^47^. Inside each magnetic layer the magnetic coupling is ferromagnetic. It is interesting that the opening of the Dirac gap at the DP is observed for this AFM TI (MnBi_2_Te_4_) up to room temperature despite that the Neel temperature is of 24.2 K^32,33^.

As to Gd-doped TIs, in ref.^40^ the magnetic properties for Bi_2(1−*x*)_Gd_2*x*_Te_3_ were analyzed theoretically and experimentally. It was shown that their magnetic structure consists of quintuple layers which can be magnetically characterized by an AFM zig-zag Gd-Te-Gd chains oriented along the c-axis. The magnetic coupling is characterized by the superexchange interaction between Gd^3+^ ions mediated via Te atoms and has AFM character below a temperature of 4.5 K. It correlates with a theoretical analysis of magnetic coupling suggested for magnetic topological insulators GdBiTe in ref.^39^ and with the interpretation of the magnetic properties of the Gd-doped TIs in refs^29,36,38,43,48^. At the same time, in each quintuple layer a non-direct FM coupling between Gd^3+^ ions is assumed. A virtual excitation from the 4*f* to the 5*d* states leads to the *f* − *f* interaction through the *d* − *f* exchange caused by the wave function overlapping between the neighboring Gd atoms^39,40^. Thereat, a weak induced antiferromagnetic ordering between Te *p*- and Gd *d*-states can stabilize the FM ordering between the nearest-neighbor Gd atoms via the *f* − *d* exchange interaction^29,36,38,39,43,48,49^.

We assume that at a higher temperature (above the bulk AFM/PM transition) the bulk AFM coupling is suppressed and only magnetic coupling inside the surface magnetic layer can develop (when the local magnetic moments at the Gd^3+^ site can be coupled via the TSS). For comparison, the existence of a bulk AFM/PM transition for RE-doped TIs was also shown in refs^29,48,49^. Wherein, the surface-sensitive XMCD measurements for the RE-doped TIs demonstrate the presence of a weak, but still distinguishable surface magnetic moment up to temperature 200–250 K^36,49^ that can testify to the surface magnetic coupling developed in these compounds at elevated temperatures. At the same time, for one EuS layer on top of TI Bi_2_Te_3_^26,27^ the surface Curie temperature reaches room temperature, while for bulk EuS the Curie temperature is 17 K. This enhancement of transition temperatures was proposed to take place due to the inclusion of the TSS in the surface magnetic coupling^27^.

Figure 5b shows the more detailed magnetic field dependences of the effective magnetization measured by SQUID at temperatures between 2 K and 100 K (taken as a more detail part from those presented in Fig. 5a with 20 times more sensitive scale). Similar independent measurements for another sample (sample 2) are presented in Supplementary Fig. 5Sb. From the SQUID data, a weak hysteresis loop is clearly visible which develops at temperature between 30 and 100 K. For temperatures of 2 K and 10 K the hysteresis loop width is much smaller, since these temperatures are close to the bulk AFM/PM transition. We associate the weak hysteresis with a magnetic contribution from the surface. The reduction of the hysteresis at temperatures closer to the bulk transition may indicate a scattering/decay channel between the magnetic moment at the surface and the bulk that destroys the TSS mediation of the magnetic coupling.

Regarding the possibility of distinguishing the surface-derived magnetic component, we have to note that the SQUID measurements presented in ref.^50^ for the system with one magnetically-ordered septuple layer of MnBi_2_Se_4_ grown by MBE on top of pristine TI Bi_2_Se_3_ showed a well-distinguished hysteresis loop measured by SQUID at temperature 4 K. Thereat, a very weak, but distinguished hysteresis loop was also observed at 300 K. These results confirm that SQUID is in principally sensitiv enough to resolve a signal from a surface magnetic layer. The surface magnetization in this system was also confirmed by corresponding surface-sensitive XMCD measurements presented in ref.^50^.

**Figure 7:**
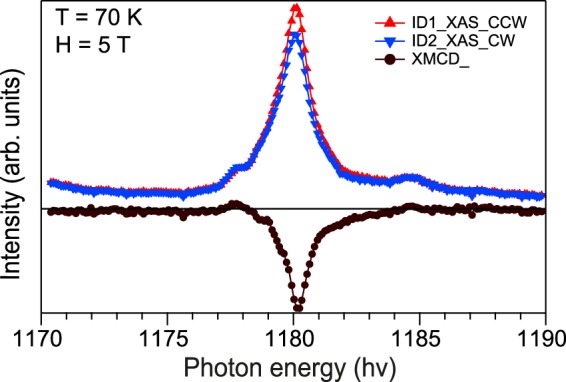
Gd M_5_ XMCD spectrum measured for Bi_1.09_Gd_0.06_Sb_0.85_Te_3_ at a temperature of 70 K under an applied magnetic field of 5 T. In the upper panel the XAS spectra at the Gd M_5_ absorption edge measured under photoexcitation by circularly polarized SR of opposite helicity are shown.

Surface-sensitive XAS and XMCD spectra acquired at the Gd M_5_ edge of Bi_1.09_Gd_0.06_Sb_0.85_Te_3_ are presented in Fig. 7. It demonstrates a pronounced asymmetry in the XMCD signal intensity under photoexcitation by circularly polarized SR of opposite helicity which is related to the existence of distinguishable surface magnetic moment at a temperature of 70 K. The XMCD spectra were measured under an applied magnetic field of 5 T perpendicular to the surface. The results of the XMCD measurements published in the literature for Gd-doped TIs^36^ and other RE-doped TIs^49^ demonstrate similar behavior and show the XMCD signal to be associated with the existence of a distinguishable surface magnetic moment up to a temperature of 200–250 K. We note here that the lineshapes of both XAS and XMCD spectra are well explained by the 3*d*^9^4*f* ^8^ multiplets for trivalent Gd ions, which again signifies the isoelectronic substitution of magnetic ions.

We associate this surface magnetism with the surface magnetic coupling where the magnetic interaction mediated by the TSS plays a significant role. We assume that this type of magnetic coupling is determined by spin-dependent hybridization of the Gd *d*, *f* states (occupied and unoccupied) with the Te *p* states and the TSS. This hybridization induces a magnetic moment at the Te *p* states and the TSS that contributes to the formation of an effective surface magnetic moment. While, one can assume that at low concentration of Gd-atoms a role of the contribution of the hybridization with the Te *p* states in the formed magnetic coupling decreases, and interaction with uniformly redistributed TSS can mainly contribute to the magnetic coupling between the Gd ions. Moreover, the hybridization-like coupling between the Gd *d*, *f* states and the TSS can be enhanced by the spin susceptibility related to the inverted band structure in TI, which is directly related to the TSS creation. At the same time, because the CB and VB states do not cross the E_F_ (see Figs 2 and 3) their contribution to the magnetic coupling formation at the surfce also decreases. As a result, the local spin at the Gd ions can effectively interact mainly via the TSS. At least, contribution of such kind magnetic interaction increases significantly. At the same time, we cannot exclude the contribution of the Van Vleck mechanism derived from the influence of the half occupied Gd 4*f* core level which can increase due to high spin susceptibility (as it was noted in ref.^51^). In addition, in ref.^18^ it was shown that when the E_F_ is inside the gap, the contribution of the RKKY coupling becomes diminished and a role of the Van Vleck magnetic interaction (and other mechanisms not related to the interaction via itinerant electrons) increases significantly.

The developed surface magnetic coupling mediated by the TSS is able to split the upper and lower DC states and open the gap at the DP which is observed in surface-sensitive photoemission spectra measured at temperatures 17, 55 and 300 K (Figs 3, 4 and Supplementary Fig. 3S). Since the spin structure of the TSS is not significantly affected by temperature, it is assumed that this type of the surface magnetic ordering is maintained up to higher temperatures. Other type of possible long-range magnetic coupling should be destroyed or at least strongly reduced at such elevated temperatures.

**Figure 8:**
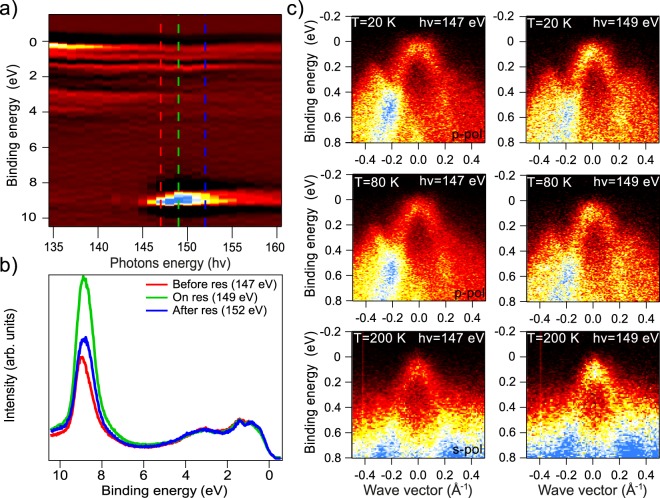
(**a**) Dependence of intensity of the Gd 4*f* states and the TSSs on the photon energy in the region of the edge Gd 4*d* − 4*f* of the photoexcitation transition. (**b**) The Gd 4*f* core level photoemission spectra measured at photon energy on-resonance (*hν* = 149 eV), off-resonance (*hν* = 147 eV) and slightly above the resonance (152 eV). (**c**) The ARPES dispersion maps showing the contributions of the lower DC states and the upper VB states which were measured at photon energy on-resonance (149 eV) and off-resonance (147 eV) at different temperatures (20 K, 80 K and 200 K) demonstrating an enhancement of the TSS intensity at photon energy corresponding to the Gd 4*d* − 4*f* resonance.

An indicator for the presence of hybridization between the Gd 4*f* states and the TSS can be a resonant increase in the intensity of the DC states at the photon energy corresponding to the edge of the Gd (4*d* − 4*f*) photoexcitation. Figure 8a shows comparable changes in the intensity of the Gd 4*f* photoemission core level peak, the TSS and the VB states. One can clearly see a strong enhancement of the Gd 4*f* peak intensity at the Gd (4*d* − 4*f*) resonance (*hν* = 149 − 150 eV). Figure 8b plots the corresponding photoemission spectra measured at photon energy of 147, 149 and 152 eV demonstrating an increase in the intensity of the Gd 4*f* peak at the edge of the Gd (4*d* − 4*f*) resonance. This resonant increase in the Gd 4*f* peak intensity should be also followed by a resonance increase in all Gd *f*-derived contributions to the VB states and the TSS which may appear as a result of hybridization of corresponding states. Figure 8c demonstrates the ARPES dispersion maps in the region of the lower DC states (and upper VB states) measured at temperatures of 20 K, 80 K and 200 K at a photon energy of 149 eV (on-resonance) and 147 eV (off-resonance). The photoemission intensity in the presented ARPES dispersion maps were normalized by the total integrated intensity over the ARPES maps. One can clearly see the relative increase in the lower DC intensity in the ARPES dispersion maps at a photon energy of 149 eV corresponding to the Gd (4*d* − 4*f*) resonance, in correlation with an increase in the intensity of the Gd 4*f* core level PE peak. This is observed for all measured temperatures.

This strongly suggests that Gd *f* states are hybridized with the DC. Such hybridization is a requisite for the the possibility of magnetic coupling between the Gd ions via the TSS at elevated temperatures. Thereat, this hybridization-like coupling can be enhanced by the spin susceptibility related to the inverted band structure in TI which is followed by the TSS creation. It provides a direct coupling between the Gd ions and the TSS, which is experimentally confirmed by the increase in the TSS intensity at the edge of the Gd (4*d* − 4*f*) photoexcitation transition. On the other hand, it can also be associated with the formation of the Van Vleck-like magnetic coupling between the half-filed Gd 4*f* states at different Gd ions due to increased spin susceptibility in such compounds. However, this issue requires more detailed study.

The fact that the Gd 4*f* core level photoemission spectrum (Fig. 8b) does not display the additional oxygen-derived components allows to exclude any possible influence of Gd_2_O_3_ (which is also AFM with an ordering temperature of 1.6 K, see^36^) on the formation of the AFM coupling in the system under study.

**Figure 9:**
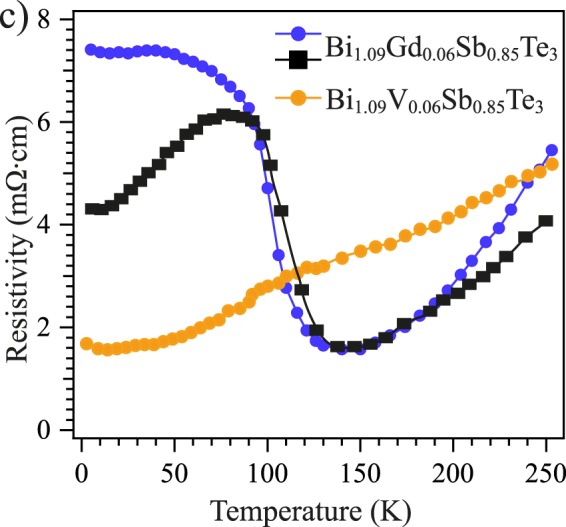
Electrical resistivity temperature dependences measured for Bi_1.09_Gd_0.06_Sb_0.85_Te_3_ (black and blue curves) and Bi_1.09_V_0.06_Sb_0.85_Te_3_ (orange curve) at temperatures between 2 and 250 K.

Figure 9 shows the temperature dependence of the electrical resistance measured for Bi_1.09_Gd_0.06_Sb_0.85_Te_3_ between 2 K and 250 K for two different samples with slightly different localization of the Dirac gap relative to E_F_. For the first sample it can be seen that up to 100 K the magnitude of resistance is approximately constant, which indicates the mid-gap semiconducting behavior with E_F_ localized within the gap. It confirms that the E_F_ is located in the Dirac gap area, and there is no carrier-dependent channel influence related to the VB or CB states which could reduce the electrical resistance. For the second sample the electrical resistance at temperatures below 50 K is lower than that of the first sample. It can be due to some initial shift of the Dirac gap relative to E_F_ followed by an appearance of some shunting charge currents. With increasing temperature, the location of E_F_ shifts to the mid-gap position with a possible simultaneous increase of the Dirac gap that is followed by a corresponding depletion of the shunting charge currents. Nevertheless, both samples demonstrate high resistance indicating the mid-gap E_F_ localization in the temperature range between 50 and 100 K. It is interesting that temperature dependence of the electrical resistance for the second sample correlates with the change in the width of the weak hysteresis loop observed in SQUID measurements up to temperature of 100 K (Fig. 5b). These observations support the assumption of the surface Dirac-fermion-mediated magnetic coupling (via the TSS) developed in Bi_1.09_Gd_0.06_Sb_0.85_Te_3_ manifesting itself in a weak hysteresis loop in the SQUID measurements, by the mid-gap semiconducting behavior of electrical resistance and the opening gap at the DP in the ARPES measurements. At temperatures above 100 K the electrical resistance first drops sharply and then increases again between 150 and 250 K demonstrating a metallic-like slow growth characteristic of TIs with low carrier density and small gap semiconducting behavior^42,52^.

Figure 9 demonstrates also, for comparison, the temperature dependence of the electrical resistance measured for Bi_1.09_V_0.06_Sb_0.85_Te_3_ between 2 and 250 K (see the orange curve). In this case, it shows a rather closer to the metallic-like behavior with slow growth with temperature from 2 K to 250 K.

## Possibility of manipulation of the surface magnetization by SR

**Figure 10:**
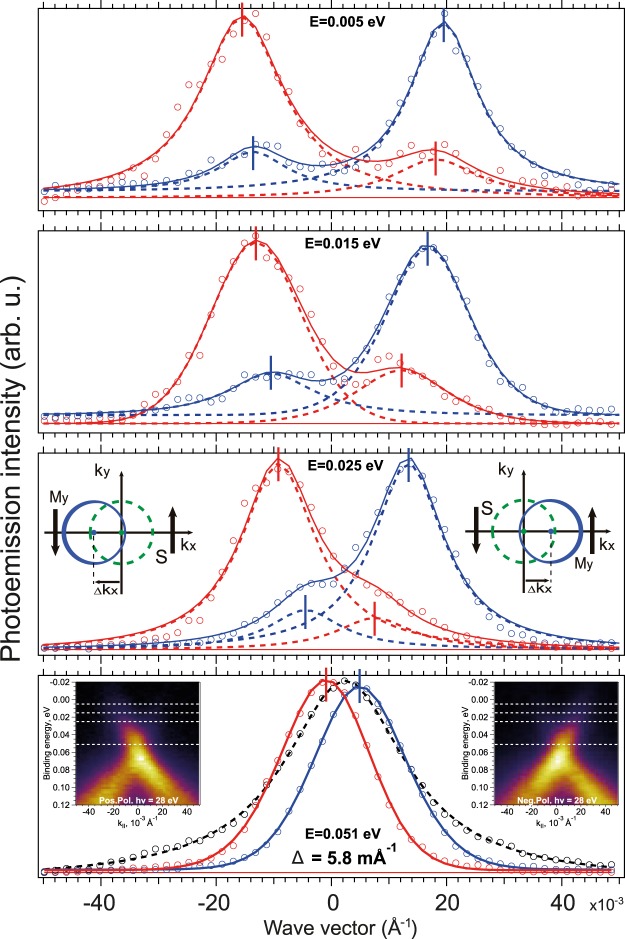
Experimentally observed MDC profiles of the DC states measured for Bi_1.09_Gd_0.06_Sb_0.85_Te_3_ at the DP demonstrating the *k*_∥_-shift between the MDC profiles under photoexcitation by circularly polarized SR of opposite helicity. The corresponding ARPES dispersion maps are shown in insets in the bottom. The schematic presentations of the relation between the asymmetry in the DC state intensity asymmetry under photoexcitation (mappings at the E_F_), the sign of the *k*_∥_-shift of the DC states, and the direction of the induced in-plane magnetic field.

After analysis of the presented data the question arises, if the surface magnetic coupling mediated by the topological DC surface states, could it be manipulated by changing the occupation of the DC states, for instance, under non-equal photoexcitation of the opposite branches DC states with opposite momentum and spin orientation? In refs^34,35^ it was shown that under photoexcitation by SR of different polarization the in-plane and out-of-plane magnetization can be induced due to non-equivalent photoexcitation of the DC states with opposite momentum and spin orientation. In accordance with refs^35,53^ such non-equivalent photoexcitation is followed by the corresponding imbalance in the generation of the holes with opposite momentum and spin orientation and the formation of a corresponding uncompensated spin accumulation. This uncompensated spin accumulation is transferred via spin-torque effect into the local in-plane and out-of-plane magnetization of the diluted magnetic ions^34,35,53^. Because the circularly polarized light induces a magnetization along the light incidence, the induced magnetization should have in-plane and out-of-plane components determined by the uncompensated spin accumulation and the induced rotation of the magnetic moment. The changes in the out-of-plane component of magnetization can be indicated by the corresponding modification of the Dirac gap in dependence on the non-equal photoexcitation of the states of the opposite DC branches^35^. The induced in-plane magnetization should manifest itself as the *k*_∥_-shift between the position of the DC states in ARPES dispersion maps or the corresponding MDC-profiles measured under photoexcitation by SR of opposite helicity^35,53^. Because spin is locked perpendicular to momentum for the TSS, the induced in-plane magnetic component is oriented perpendicular to the direction of the realized asymmetry in the DC states under photoexcitation. It is schematically presented in insets in Fig. 10, where the incident SR is oriented along the *k*_*y*_-direction. As a result, it followed by the *k*_∥_-shift of the DC states along the observed asymmetry in the DC states^34,35^.

To analyze the SR-induced *k*_∥_-shift of the DC states Fig. 10 demonstrates the Momentum Distribution Curve (MDC) profiles measured under photoexcitation by SR of opposite helicity at different E_B_ between the DP and E_F_. The corresponding APRES dispersion maps are shown in insets in the bottom. Comparison between the ARPES dispersion maps, measured at opposite circular polarizations of SR, and corresponding MDC profiles shows the reversal on their intensity asymmetries of the upper DC. It is followed by the corresponding *k*_∥_-shift between the DC state maxima in the cases of photoexcitation by SR of opposite circular polarization. Schematic plots of the relation between the DC state intensity asymmetry in the *k*_*x*_, *k*_*y*_-mappings at E_F_, the sign of the *k*_∥_-shift, and the direction of induced in-plane magnetic field are also shown as insets in Fig. 10. It means that the *k*_∥_-shift of the DC states can be an indicator of the in-plane component of the SR-generated magnetization^34,35,54^. Figure 10 shows the *k*_∥_-shift between the MDC measured at opposite helicity of SR at different E_B_ along the upper DC (see horizontal white lines).The experimentally estimated value of the *k*_∥_-shift at the DP is of about 5.8 × 10^−3^ Å^−1^. Along the upper DC the *k*_∥_-shift has approximately constant value of about 4 − 5 × 10^−3^ Å^−1^ with a decrease close to E_F_. Some distortion of the DC structure and the corresponding *k*_∥_-shift near E_F_ (noted in ref.^55^) can be related to the zero-bias spin-polarized current accompanying the SR generated uncompensated spin accumulation.

Simultaneously as mentioned above, photoexcitation by SR with a non-zero helicity is also accompanied by a decrease in the splitting between the lower and upper DCs to a value of 22–23 meV (on average), which is less than the splitting value of 27 meV observed under photoexcitation by linearly-polarized SR (see Fig. 3). It can testify to the change of the out-of-plane component of magnetic moment induced by SR^34,35^. The decrease of the DC state splitting at the DP and the appearance of the *k*_∥_-shift can be related to some rotation of the Gd^3+^ magnetic moments induced by circularly polarized SR relative to the spontaneous out-of-plane orientation towards the direction of the SR incidence.

The observed changes of the in-plane and out-of-plane components of the magnetic moments are induced by circularly polarized SR due to non-equivalent depopulation of the DC states with opposite momentum under photoexcitation. Therefore, they can be an indicator of the modification of the Dirac-fermion-mediated magnetic coupling by SR.

### Bi_1.09_V_0.06_Sb_0.85_Te_3_

It is interesting to compare the features of the electronic structure measured by ARPES and the magnetic properties of Bi_1.09_Gd_0.06_Sb_0.85_Te_3_ with those characteristic of TM-doped magnetic TI Bi_1.09_V_0.06_Sb_0.85_Te_3_, which exhibits an analogous stoichiometry and geometric structure. For this type of TI the DP (and corresponding Dirac gap) is also located at E_F_. Although in this case the substitution of Bi^+3^ by V-ions is accompanied by the introduction of additional charge carriers derived from the V 3*d* impurity band, which is expected to be located at E_F_^53,56,57^.

**Figure 11:**
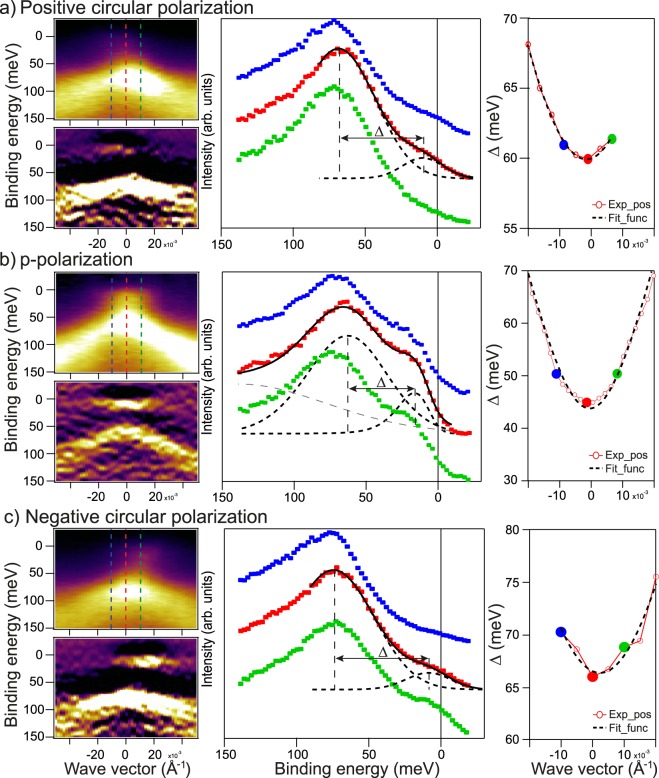
Left column - the ARPES dispersion maps measured Bi_1.09_V_0.06_Sb_0.85_Te_3_ at temperature 55 K and photon energy of 28 eV under photoexcitation by SR of different polarizations (linear p-polarization – (**b**) and circular ones of opposite helicity - (**a**,**c**)). Both the ARPES dispersion maps are presented in the N(E) and *d*^2^*N*/*dE*^2^ forms for better visualization of the electronic structure near the DP. The middle column - the corresponding EDC-profiles measured directly at the Γ-point (*k*_∥_ = 0) and at opposite *k*_∥_ relative to the Fermi level with decomposition on the spectral components (red lines). Right column – the dependence of the estimated splitting between the lower and upper DC states on *k*_∥_.

Figure 11 shows the ARPES dispersion maps (left column) measured for Bi_1.09_V_0.06_Sb_0.85_Te_3_ at a temperature of 55 K under photoexcitation by linear p-polarized - (b) and circularly polarized SR of opposite helicity - (a, c) at a photon energy of 28 eV (also after corresponding *k*_*x*_, *k*_*y*_-mapping which is presented in Fig. 2c). For Bi_1.09_V_0.06_Sb_0.85_Te_3_ the DP (and the corresponding gap) is also located close to the Fermi level. For this compound the *d*^2^*N*/*dE*^2^ presentation shows a dip between the lower and upper DC states more clearly. In the middle column, the EDCs measured directly at the DP (at *k*_∥_ = 0) (red curves) and for opposite momenta relative to the Γ-point (blue and green curves) are shown with the fitting into spectral components. For the estimation of the gap value the same three methods were used as for the analysis of the data presented in Fig. 3. The results of using these methods are shown in the corresponding insets in right column in Fig. 11 by open red circles, black dashed lines and green, blue, red circles for all used polarization of SR. As before, the final value of the DC splitting at the DP was estimated as the minimal value obtained at the DP using the plotted *k*_∥_-dependence. The result of the gap estimation at the DP for Bi_1.09_V_0.06_Sb_0.85_Te_3_ gives the Dirac gap value measured under photoexcitation by linear p-polarized SR of about 44–45 meV (±3 meV). The higher splitting of the DC states at the DP compared with that observed for the Bi_1.09_Gd_0.06_Sb_0.85_Te_3_ (27 meV) may be due to the additional charge carrier doping caused by the non-isoelectric substitution of Bi by V which occurs from the additional V 3*d* impurity band^56,57^ whose states also contribute to the magnetic coupling. The *k*_*x*_, *k*_*y*_-mappings of the DC and VB states for Bi_1.09_V_0.06_Sb_0.85_Te_3_ cut at different E_B_ near the Fermi level shown in Fig. 2c also demonstrate that the VB and CB band states do not cross E_F_.

Interestingly, for this kind of V-doped TI (Bi_1.09_V_0.06_Sb_0.85_Te_3_) with the Dirac gap located near E_F_, the temperature dependence of the magnetic susceptibility (*χ*) also demonstrates a negative Weiss temperature (−4.2 K), see Supplementary Fig. 9S. It can also testify to the bulk AFM coupling at low temperatures that differs fundamentally from other V-doped TI with Dirac gap located away from E_F_, where the VB and CB states contribute to the magnetic coupling formation (see, for comparison, the magnetic susceptibility measurements for Bi_1.31_V_0.03_Sb_0.66_Te_3_ presented in Supplementary Fig. 7S).

The SQUID measurements Bi_1.09_V_0.06_Sb_0.85_Te_3_(Supplementary Fig. 8S) do not exhibit visible hysteresis at any temperature.

The temperature dependence of the electrical resistance measured for Bi_1.09_V_0.06_Sb_0.85_Te_3_ shown in Fig. 9 displays close-to-metallic behavior with a slow increase for temperatures between 2 K and 250 K. The difference to the temperature dependence observed for Bi_1.09_Gd_0.06_Sb_0.85_Te_3_ is probably caused by the shunting effect of the carriers derived from the V impurity *d*-band located at E_F_^56,57^. It leads to significant reduction of electrical resistance at low temperatures, where such a contribution would be dominant. At higher temperatures the electrical resistance reaches values comparable to Bi_1.09_Gd_0.06_Sb_0.85_Te_3_.

Figure 11 shows ARPES dispersion measured for Bi_1.09_V_0.06_Sb_0.85_Te_3_ under photoexcitation by circular polarized SR of opposite helicity – (a, c). One can clearly identify the redistribution of spectral weight between the DC branches of opposite momentum is reversed for opposite circular polarization (despite their low absolute spectral weight). In the middle column EDCs measured directly at the DP (*k*_∥_ = 0) and for opposite momenta relative to the Γ-point are plotted. For the spectra at the DP the fitting of the EDC with decomposition into spectral components are presented for all used polarization of SR. The dependences of the DC states splitting for different *k*_∥_-values are plotted in the right column (compare Fig. 3). Here, the values of the splitting between the lower and upper DC states at the DP (or the Dirac gap) as excited by circularly polarized SR can be estimated to around 60 and 65 meV respectively. Due to the significant asymmetry in the intensity of the DC state branches with opposite momentum under photoexcitation by circularly polarized SR relative to *k*_∥_ = 0 the intensity of the TSS at the DP is reduced. As a result, the measured gap value increases. It can lead to an additional error in estimating the gap value (no less than ±5 meV). Nevertheless, the increase in the DC splitting and the corresponding gap at the DP in comparison with that observed under photoexcitation by p-polarized SR is observed. The gap also becomes visible in the *d*^2^*N*/*dE*^2^ representation of ARPES maps. It is interesting that this behavior is in opposite to the case of the Gd-doped TI (Bi_1.09_Gd_0.06_Sb_0.85_Te_3_), where photoexcitation by circularly polarized SR led to decreasing of the Dirac gap.

To analyze the correlation between the SR-generated out-of-plane and in-plane magnetization component the corresponding *k*_∥_-shift between the MDCs in the ARPES dispersion maps was measured under photoexcitation using circular polarized SR of opposite helicity (Supplementary Fig. 10S). The value of the *k*_∥_-shift at the DP is of about 3.5 × 10^−3^ Å^−1^. That is half compared to Bi_1.09_Gd_0.06_Sb_0.85_Te_3_. It can testify to the lower rotation of magnetic moment for Bi_1.09_V_0.06_Sb_0.85_Te_3_. The higher value of the in-plane component of the induced magnetization for Bi_1.09_Gd_0.06_Sb_0.85_Te_3_ is accompanied by lower value of the out-of-plane component and vice versa. The overall higher value of the Dirac gap for Bi_1.09_V_0.06_Sb_0.85_Te_3_ can be related to additional influence of the impurity V *d*-band states that increase the total magnetic moment.

Taking these observations into account we cannot exclude the influence of the SR-generated magnetization under photoexcitation by p-polarized SR on the Dirac gap formation up to room temperature, too, (possibly including the effect of depopulation of the VB states). This kind of the surface magnetization should be very sensitive to the photon energy used and the details of the experiment. This very complex problem requires further research.

## Experimental inaccuracy in the Dirac gap and *k*_∥_-shift estimation

Here, we have to discuss the problem of the inaccuracy in experimental estimation of the *k*_∥_-shift of the DC states which, according to refs^34,35^, depends on the statistics of the measured MDC profiles, the number of spectral points and the one pixel size inaccuracy (of about 2 × 10^−3^ Å^−1^ in the current experiment)and was estimated to be Δ*k*_*shift*_ = 2.5 × 10^−3^ Å^−1^. While the experimentally estimated *k*_∥_-shift for Bi_1.09_V_0.06_Sb_0.85_Te_3_ and Bi_1.09_Gd_0.06_Sb_0.85_Te_3_ (3.5 × 10^−3^ Å^−1^ and 5.8 × 10^−3^ Å^−1^, respectively) are close to this level, it is enough to estimate an approximately two times higher in-plane component of magnetization induced by Bi_1.09_Gd_0.06_Sb_0.85_Te_3_.

At the same time, the finite size and the analyzer slit size can also affect the results of the evaluation of the DC state splitting at the DP, see for details ref.^35^. Supplementary Fig. 11S shows the simulation of the ARPES dispersion map and possible error in estimating the Dirac gap value at the sizes of the analyzer slit used in the current measurements. The results of the simulation show that the minimal gap value which can be reasonably distinguished by ARPES measurements at the used experimental condition is 15–20 meV. For larger “real” gap values, the resulting “measured” gap value is only increased by some meV, the detection limit under current conditions has been estimated to be a gap of 20–25 meV, which would be artificially increased to a visible gap value of 24–28 meV. In the case when the Dirac gap is located close to the E_F_, the error in the Dirac gap estimation is reduced (up two times) due to a significantly reduced contribution from the upper DC states. It means that the experimental observation of the splitting of the DC states at the DP is certainly higher than the error caused by the finite size of the analyzer slit.

At the end we would like to note that the surface Dirac-fermion-mediated magnetic coupling observed in Bi_1.09_Gd_0.06_Sb_0.85_Te_3_ and its unusual surface magnetic properties correlate with the results of refs^50,58^ devoted to study of systems with 2D surface ferromagnetism and paramagnetic bulk (for instance, monolayer of VSe_2_ on HOPG or MnBi_2_Se_4_ on Bi_2_Se_3_) characterized by unusual magnetic properties up to room temperature. Systems with 2D ferromagnetism have attracted an enhanced interest in recent years due to further development of theory of surface magnetism. They are very promising in future spintronic applications and require detailed investigations.

## Conclusion

We have shown that the electronic structure of Gd-doped topological insulator with stoichiometry Bi_1.09_Gd_0.06_Sb_0.85_Te_3_ is characterized by a gapped Dirac cone located at the Fermi Level without additional crossing of either valence or conduction band. We further demonstrate the persistence of the Dirac Gap to temperatures as high as room temperatures, which are well above the bulk magnetic transition. The temperature dependence of the magnetic susceptibility indicates a bulk AFM phase at low temperatures. As a result, the material can be considered as a magnetic topological insulator with AFM coupling below the Neel temperature (4–8 K). We assume that at temperatures higher than the bulk AFM/PM transition, a magnetic surface layer is present, where coupling between the magnetic moments located at magnetic impurities (Gd) is mediated by a surface Dirac-fermion magnetic coupling. This hypothesis is further supported by a weak hysteresis loop measured through SQUID at temperatures between 30 and 100 K as well as by XMCD measurements demonstrating a surface magnetic moment at 70 K.

This kind of magnetic coupling is determined by a spin-dependent hybridization of the Gd *d*, *f* states (occupied and unoccupied) with the topological surface state which can be enhanced by the spin susceptibility related to the inverted band structure in topological insulators. This hybridization was confirmed by resonant photoemission of the topological surface states and the valence band at the Gd edge. At the same time, a contribution derived from magnetic coupling between the half-filed Gd 4*f* states at different Gd ions due to increased spin susceptibility in such compounds (Van Vleck mechanism) cannot be excluded.

Photoexcitation by circularly polarized synchrotron radiation leads to a decrease in the gap at the Dirac point of Bi_1.09_Gd_0.06_Sb_0.85_Te_3_ and an increase for Bi_1.09_V_0.06_Sb_0.85_Te_3_. Simultaneously, it is followed by a *k*_∥_-shift of the DC states twice lower for Bi_1.09_V_0.06_Sb_0.85_Te_3_, i.e. the higher value of the in-plane component of the induced magnetization for Bi_1.09_Gd_0.06_Sb_0.85_Te_3_ is accompanied by the lower value of the out-of-plane component and vice versa. These observations can be an indicator of the induced out-of-plane and in-plane magnetizations generated by synchrotron radiation, which are associated with the rotation of the Gd^3+^ magnetic moments induced by circularly polarized. Because these effects are determined by the non-equivalent depopulation of the topological surface states with opposite momentum under photoexcitation, they can be an indicator of the modification of the surface magnetic coupling mediated by the topological surface states.

Taking into account all the observations discussed above, we can assume that Bi_1.09_Gd_0.06_Sb_0.85_Te_3_ is a very promising candidate for effective realization of the Quantum Anomalous Hall effect with a possibility to an expansion to room temperature.

## Methods

**Figure 12:**
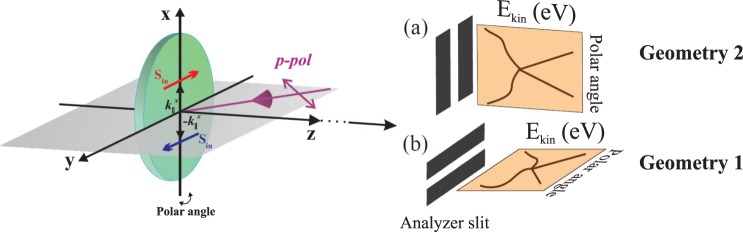
Methods section. Schematic presentation of the ARPES measurements geometries with the analyzer slits oriented along (Geometry 1) and perpendicular (Geometry 2) to the SR incidence plane.

The measurements of the ARPES dispersion maps and core level photoemission spectra were carried out at the 1^2^ end station at BESSY II (Helmholtz-Zentrum Berlin, Germany) and at the linear undulator beamline BL-1 at HiSOR^59^ (Hiroshima, Japan) using a Scienta R4000 analyzer at the incidence angle of SR of 50° relative to the surface normal. Geometry of the ARPES experiment is shown in Fig. 12 In the case of the measurements of the ARPES dispersion maps at BESSY Geometry 2 was used. For the experiment at HiSOR Geometry 1 was used.

For analysis of magnetic properties of Bi_1.09_Gd_0.06_Sb_0.85_Te_3_ and Bi_1.09_V_0.06_Sb_0.85_Te_3_ the magnetometric measurements using a Superconducting Quantum Interference Device magnetometer were carried out in Research Park of Saint Petersburg State University “Centre for Diagnostics of Functional Materials for Medicine, Pharmacology and Nanoelectronics”.

The temperature dependences of the electrical resistivity were measured in Novosibirsk State University.

The XMCD measurements were carried out at the BL23SU apparatus at SPring-8 (Japan).

The sample of Bi_1.09_Gd_0.06_Sb_0.85_Te_3_ was characterized by X-ray diffractometer (model: Ultima IV, Rigaku, Japan) with Cu K *α* radiation (*λ* = 1.5406 Å) operating at 40 kV and 30 mA at HiSOR (Hiroshima, Japan).

The single crystals of Bi_1.09_Gd_0.06_Sb_0.85_Te_3_ and Bi_1.09_V_0.06_Sb_0.85_Te_3_ were synthesized by using a modified vertical Bridgman method^60^ in Novosibirsk State University. Clean surfaces of the samples were obtained by a cleavage in ultrahigh vacuum. The base pressure during the experiment was better that 1 × 10^−10^ mbar.

## Supplementary information

Supplementary information
