# Supplementary material for: Dirac gap opening and Dirac-fermion-mediated magnetic coupling in antiferromagnetic Gd-doped topological insulators and their manipulation by synchrotron radiation
Source: Sci Rep. 2019 Mar 18;9:4813. doi: 10.1038/s41598-019-41137-w (PMC6423091; doi:10.1038/s41598-019-41137-w)
Supplement: Supplementary file 1 — Supplementary information [file 41598_2019_41137_MOESM1_ESM.pdf]

Supplementary informtion.

Dirac gap opening and Dirac-fermion-mediated magnetic coupling in antiferromagnetic Gd-doped topological insulator and their manipulation by synchrotron radiation

A.M. Shikin[1,*], D.A. Estyunin[1], Yu.I. Surnin[1], A.V. Koroleva[1], E.V. Shevchenko[1], K.A. Kokh[1,2,3], O.E. Tereshchenko[1,2,4], S. Kumar[5], E.F. Schwier[5], K.Shimada[5], T. Yoshikawa[7], Y. Saitoh[6], Y. Takeda[6], and A. Kimura[7]

[1]Saint Petersburg State University, Saint Petersburg, 198504 Russia
[2]Novosibirsk State University, Novosibirsk, 630090 Russia
[3]V.S. Sobolev Institute of Geology and Mineralogy, Novosibirsk, 630090 Russia
[4]A.V. Rzhanov Institute of Semiconductor Physics, Novosibirsk, 630090 Russia
[5]Hiroshima Synchrotron Radiation Center, Hiroshima University, Hiroshima 739-0046, Japan
[6]Materials Sciences Research Center, Japan Atomic Energy Agency, Hyogo 679-5148, Japan
[7]Department of Physical Sciences, Graduate School of Science, Hiroshima University, Hiroshima 739-8526, Japan
[*]ashikin@inbox.ru
a.shikin@spbu.ru

1 Energy shift of the DC states and the Dirac point for $Bi_{1.09}Gd_{0.06}Sb_{0.85}Te_3$ towards higher E_B with time in the ARPES dispersion map

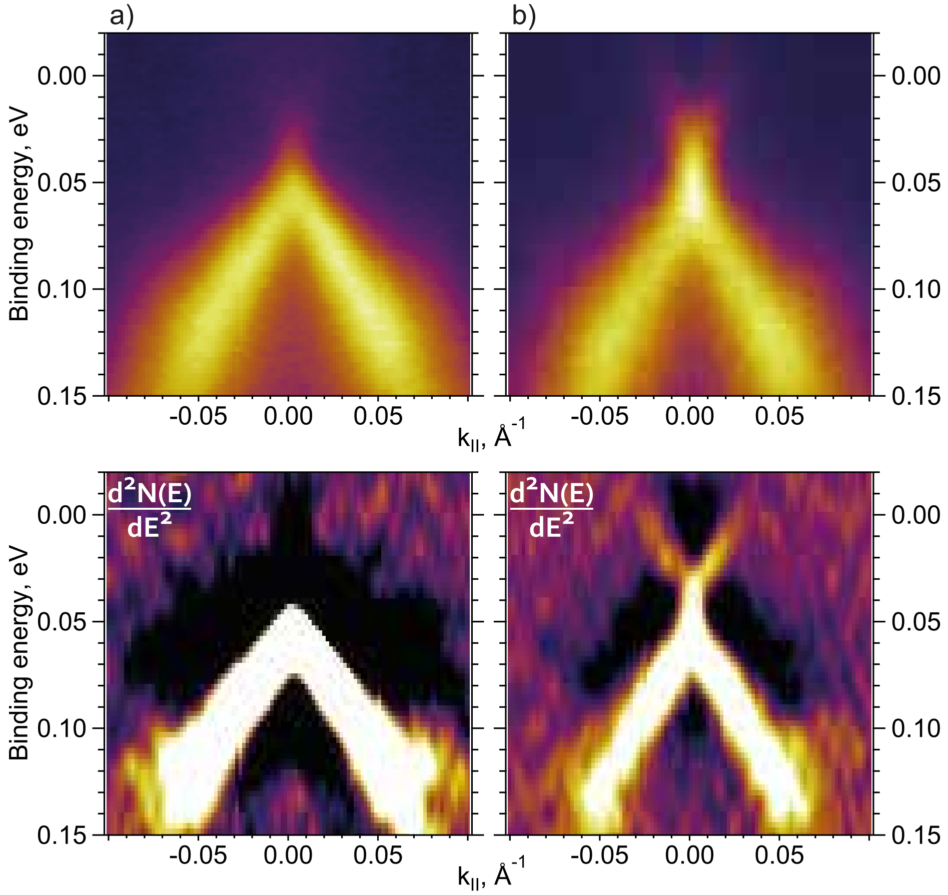

Figure 1. **S** The ARPES dispersion maps for the DC states measured for $Bi_{1.09}Gd_{0.06}Sb_{0.85}Te_3$ at a temperature of 55 K and a photon energy of 28 eV exactly after the cleavage – (a) and after exposition in atmosphere of residual gases in the experimental chamber during 4 – 5 hours. The energy shift towards higher E_B and partial occupation of the upper DC states are visible. Below the ARPES dispersion maps in the form d^2N/dE^2 are presented for better visualization.

2 Structure of the upper edge VB states for $Bi_{1.09}Gd_{0.06}Sb_{0.85}Te_3$ measured at different photon energy

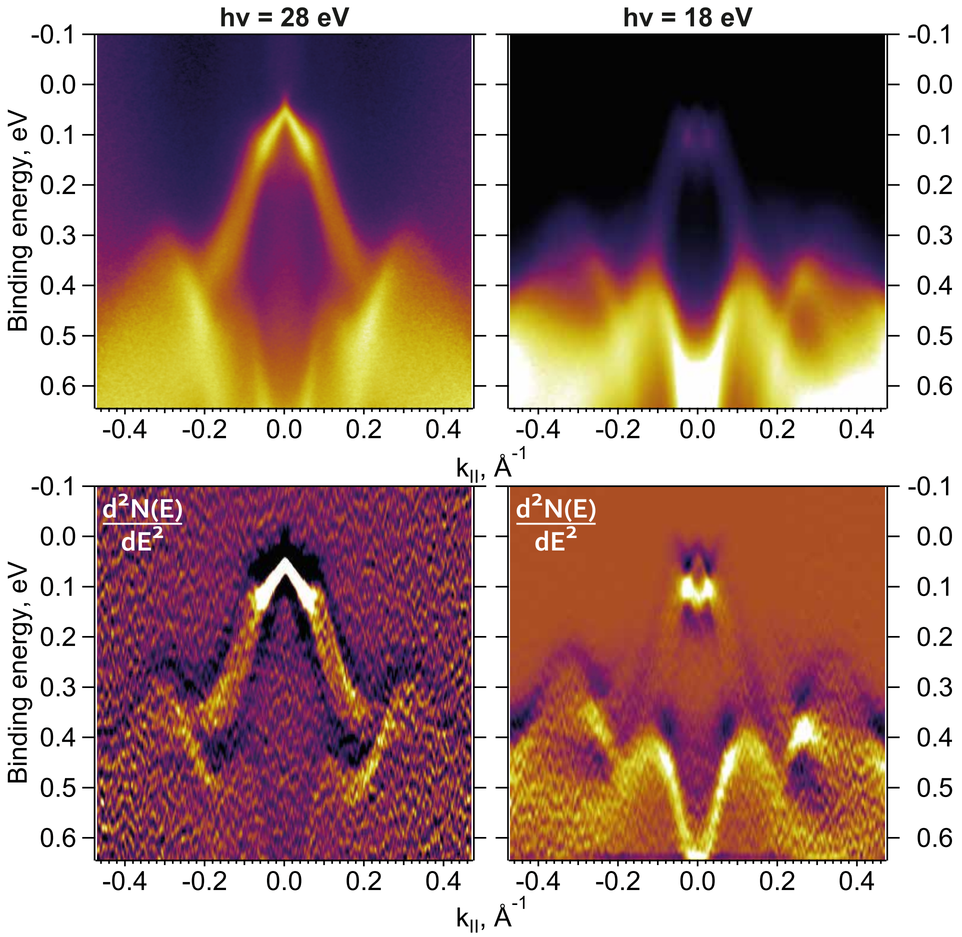

Figure 2. **S** The ARPES dispersion maps for the TSS and the upper VB states measured for $Bi_{1.09}Gd_{0.06}Sb_{0.85}Te_3$ at a temperature of 55 K and a photon energy of 28 eV and 18 eV. Below the ARPES dispersion maps in the d^2N/dE^2 form are presented for better visualization.

3 ARPES dispersion map and estimation of the gap open at the Dirac point measured at low temperature

Fig. 3S shows the results of the estimation of the gap opening at the DP at low temperature (17 K) for $Bi_{1.19}Gd_{0.06}Sb_{0.75}Te_3$ at a photon energy of 30 eV. In comparison with spectra shown in Fig.4 of the main text, the presented spectra were measured at the wider angle mode (30°). The estimation of the gap at the DP in this case gives slightly larger value of the gap (about 35 meV). Thereat, the dip between the upper and lower DC states is more visible, and the contributions of the VB states and the TSS at the DP can be separated. The upper edge of the VB states is located at the DP at the E_B of about 60 meV. For the TSS it is located at the E_B 40 meV.

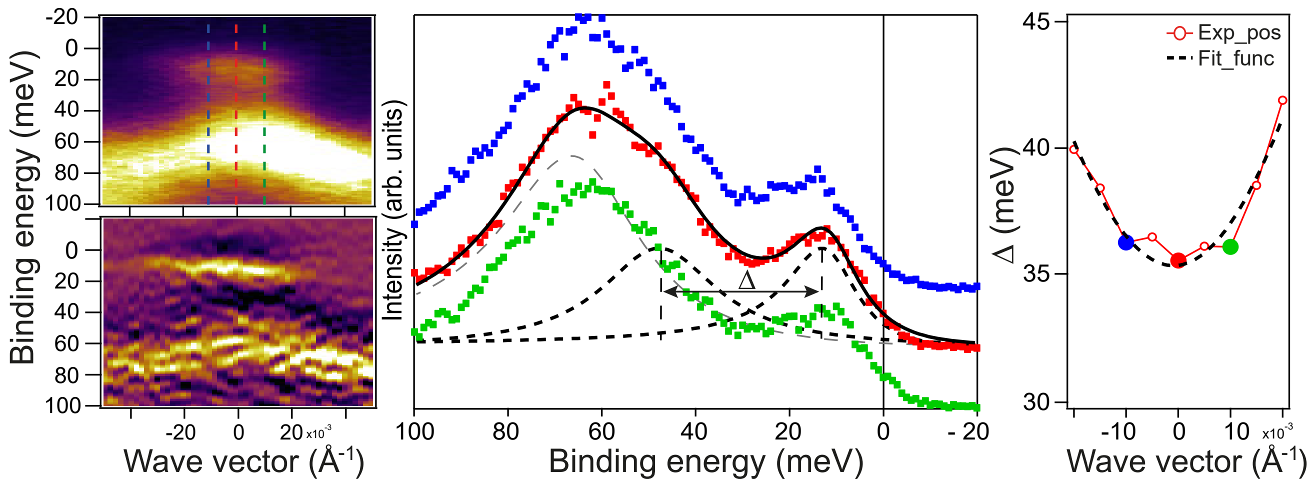

Figure 3. S Left column - ARPES dispersion maps measured for $Bi_{1.19}Gd_{0.06}Sb_{0.75}Te_3$ at a temperature of 17 K and a photon energy of 30 eV under photoexcitation by linear p-polarized SR. ARPES dispersion maps are presented both in the N(E) and d^2N/dE^2 forms for better visualization of the electronic structure near the DP. Middle column - corresponding EDC-profiles measured directly at the *Gamma*-point ($k_{\|} = 0$) and at opposite $k_{\|}$ relative to the Fermi level with decomposition on the spectral components (red lines). Right column – the dependence of the estimated splitting between the lower and upper DC states on $k_{\|}$ estimated by three methods described in the main text of the manuscript.

4 Structure of the DC states and upper VB states for $Bi_{1.09}Gd_{0.06}Sb_{0.85}Te_3$ presented in the circular dichroism signal dispersion map under photoexcitation by circularly polarized SR of opposite helicity

Supplementary Fig.4S shows the result of subtraction of the ARPES dispersion maps measured under photoexcitation by circularly polarized SR of opposite helicity (so called circular dichroism (CD) signal). Because photoexcitation by SR of opposite helicity is determined by the difference in the photoexcitation matrix elements for the DC states with opposite spin orientation, the CD signal can be an indicator of localization of the states with different spin orientation.

In this relation, on the one hand, we can distinguish in Supplementary Fig.4S the topological DC states of the lower and upper DCs with the inverse spin orientation. These states are located in the energy region between the E_F and E_B 0.08 – 0.09 eV. On the other hand, in the region of the lower DC states some mixing with the upper edge states of the VB, which are also spin polarized, can take place. The EDC profiles in the difference CD signal measured at opposite $k_{||}$ (see the vertical lines in Supplementary Fig.4S.a) confirm the spin structure inversion of the DC states relative to the Γ point ($k_{||} = 0$) that is typical for the DC states. I.e. the states in the region between 0.08 – 0.09 eV and the E_F can be ascribed to the upper and lower DCs with some mixing of the lower DC states with the VB states

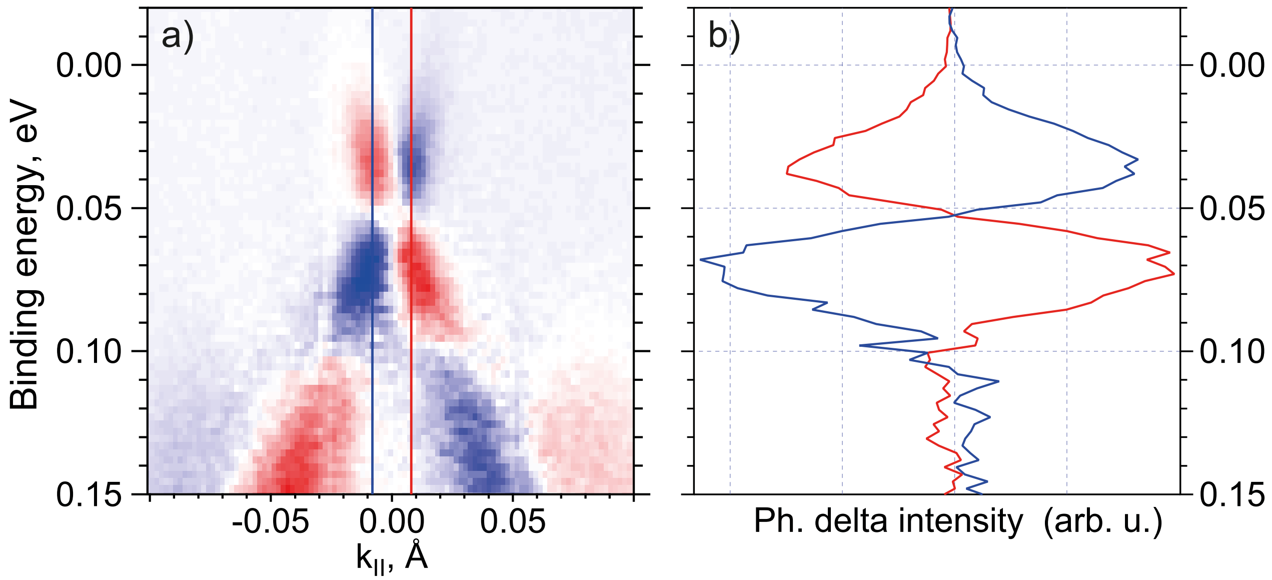

Figure 4. S (a) – the dispersion map obtained as result of subtraction between the ARPES dispersion maps for $Bi_{1.09}Gd_{0.06}Sb_{0.85}Te_3$ under photoexcitation by circularly polarized SR of opposite helicity (the circular dichroism signal) at a photon energy of 28 eV. (b) - the EDC profiles in difference CD signal measured at opposite $k_{||}$ (marked by the vertical lines in (a)) which confirm the spin structure inversion of the DC states relative to the Γ point ($k_{||} = 0$) characteristic for the DC states.

5 SQUID measurements for $Bi_{1.09}Gd_{0.06}Sb_{0.85}Te_3$ at different temperatures (sample 2)

Fig.5S shows the dependences of the magnetization in $Bi_{1.09}Gd_{0.06}Sb_{0.85}Te_3$ (sample 2) as a function of the applied out-of-plane magnetic field (B//c) measured by SQUID at different temperatures from 2 K to 300 K. The experimental curves demonstrate behavior similar to sample 1 presented in the main text with gradual saturation achieved at 2 T.

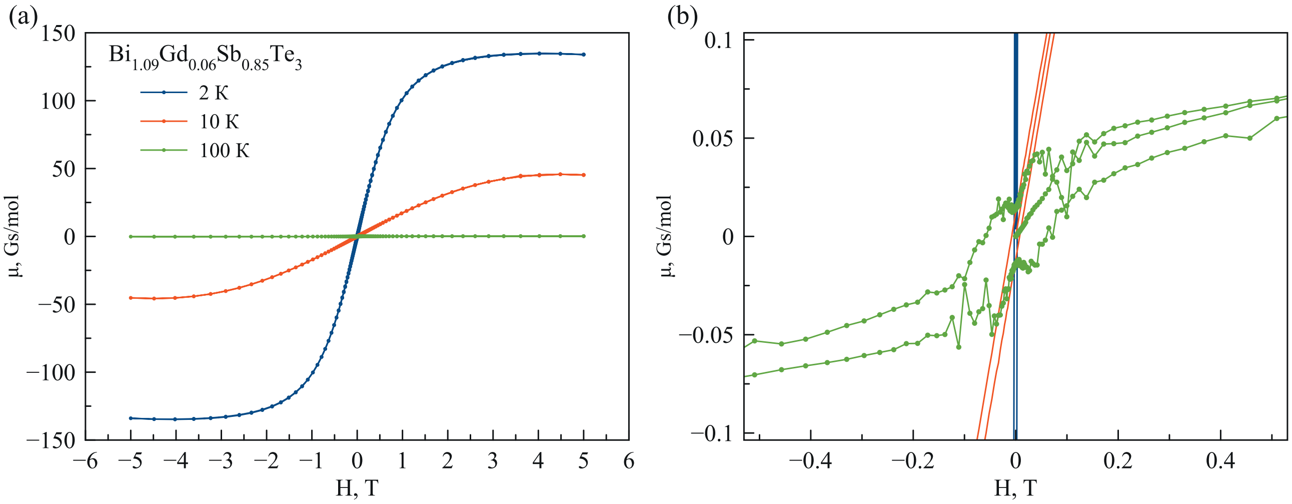

Figure 5. S (a) - the magnetic field dependences of the effective magnetization for $Bi_{1.09}Gd_{0.06}Sb_{0.85}Te_3$ (sample 2) measured at different temperatures (2 K and 100 K) with an external magnetic field applied perpendicular the surface. (b) - detailed magnetic field dependences of the effective magnetization for $Bi_{1.09}Gd_{0.06}Sb_{0.85}Te_3$ at temperatures between 2 K and 100 K, taken from (a) for lower values of the applied magnetic field.

6 The temperature dependence of the magnetic susceptibility for $Bi_{1.09}Gd_{0.06}Sb_{0.85}Te_3$ (sample 2)

The temperature dependence of the magnetic susceptibility under an applied out-of-plane field of 5T in the range of temperatures between 2 K and 300 K is presented in Fig.6S.

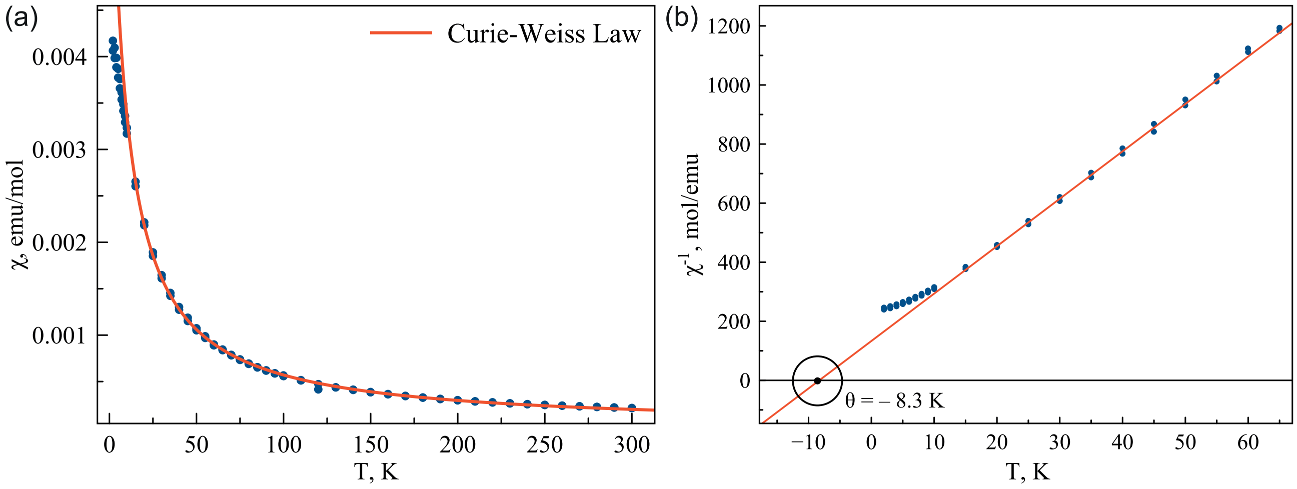

Figure 6. S (a) – the temperature dependence of the magnetic susceptibility (χ) measured for $Bi_{1.09}Gd_{0.06}Sb_{0.85}Te_3$ (sample 2) with an external magnetic field (5 T) applied perpendicular the surface with approximation by the Curie-Weiss law at low temperatures (red lines). (b) - the temperature dependence of the inversed magnetic susceptibility $1/(\chi)$ with corresponding approximation at low temperatures (red lines) showing a negative values of the Weiss temperature ($\Theta = -8.3$ K).

7 The temperature dependence of the magnetic susceptibility for $Bi_{1.31}V_{0.03}Sb_{0.66}Te_3$

Fig.7S demonstrates, for comparison, the temperature dependence of the magnetic susceptibility (χ) and the inverse temperature dependence of the magnetic susceptibility $1/(\chi)$ measured for classical V-doped TI with stoichiometry $Bi_{1.31}V_{0.03}Sb_{0.66}Te_3$ which is characterized by a positive value of the Weiss temperature ($\Theta = 2.4$ K). The dependence demonstrates the behavior characterized by the FM/PM transition traditional for the TM-doped TIs.

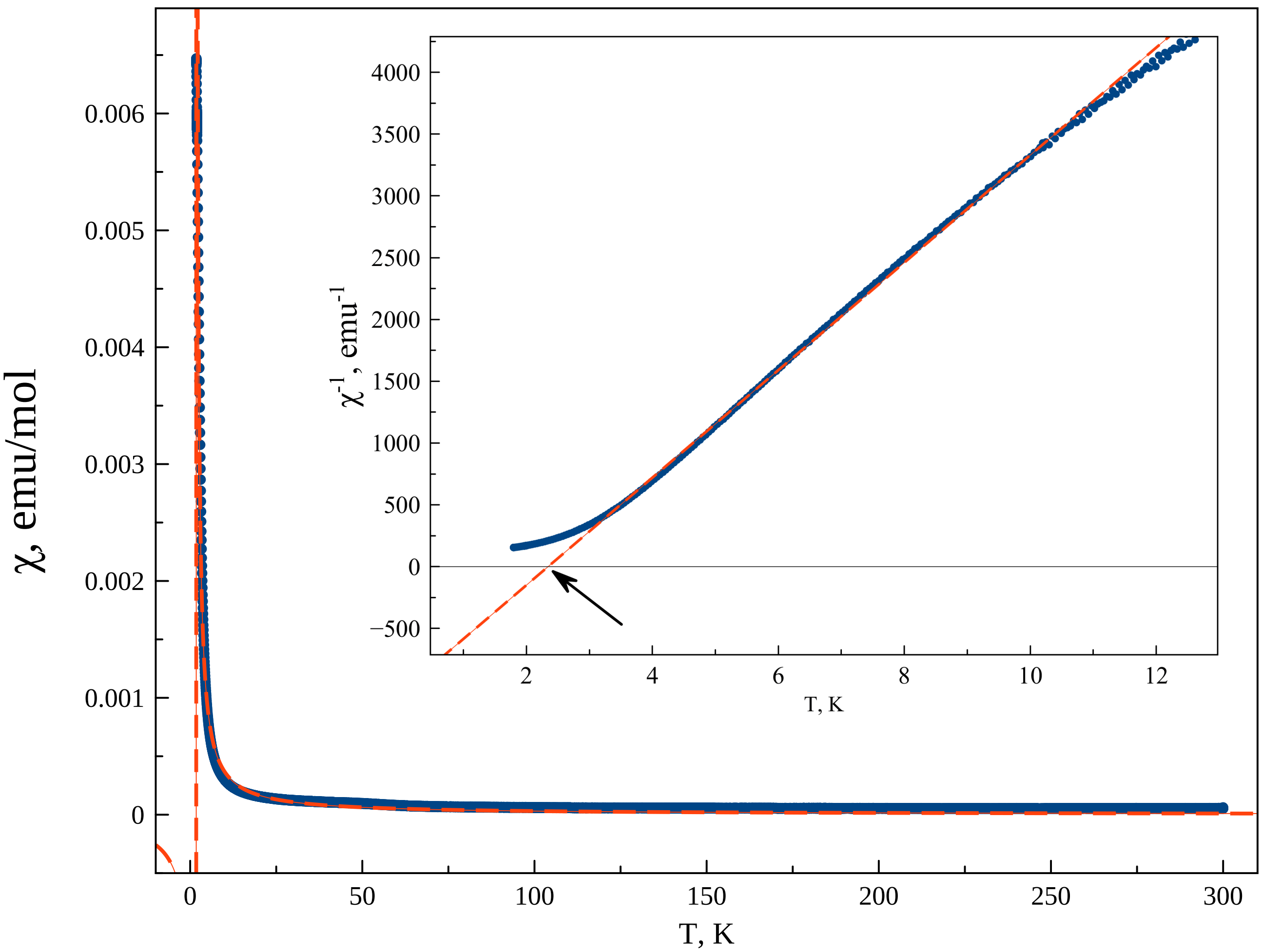

Figure 7. S The temperature dependence of the magnetic susceptibility (χ) measured for $Bi_{1.31}V_{0.03}Sb_{0.66}Te_3$ with an external magnetic field applied perpendicular the surface with approximation by the Curie-Weiss law at lower (red line). Inset - the inverse temperature dependence of the magnetic susceptibility $1/(\chi)$ and its linear approximation at low temperatures (red line) showing a positive value of the Weiss temperature ($\Theta = 2.4$ K).

8 SQUID measurements for $Bi_{1.09}V_{0.06}Sb_{0.85}Te_3$ at different temperatures

Fig.8S.a shows the dependences of the sample magnetization per mole as a function of an applied out-of-plane magnetic field (B//c) measured for $Bi_{1.09}V_{0.06}Sb_{0.85}Te_3$ by SQUID at temperatures 2 K and 100 K. The measured dependences demonstrate a gradual character with saturation achieved at 2 T, as in the case of the $Bi_{1.09}Gd_{0.06}Sb_{0.85}Te_3$. Fig.8S.b presents more detailed magnetic field dependences of the effective magnetization for $Bi_{1.09}Gd_{0.06}Sb_{0.85}Te_3$ at temperatures of 2 K and 100 K (taken as a part of Fig.8S.a) for lower scale of the applied magnetic field. For this compound we cannot already distinguish a visible hysteresis loop (as in the case of the Gd-doped TI). Only S-like behavior of the magnetization curve characteristic of paramagnetic phase is observed. It means that for the V-doped TI even with similar stoichiometry and localization of the DP close to the E_F the Dirac-fermion-mediated coupling cannot be clearly distinguished due to the shunting effect of magnetic coupling via the impurity d-band located at the E_F[1].

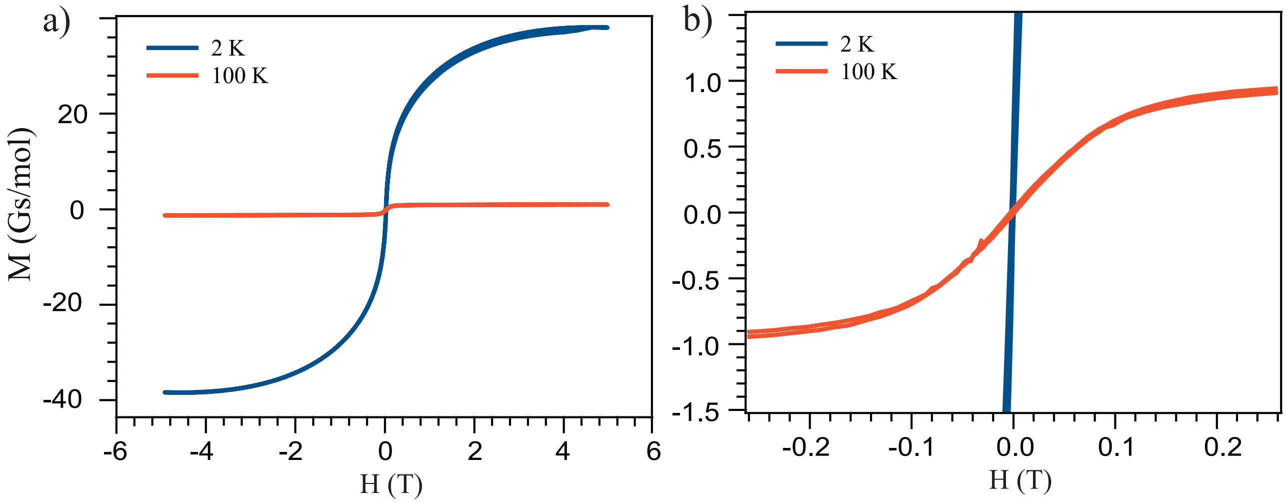

Figure 8. **S** The magnetic field dependences of the effective magnetization for $Bi_{1.09}V_{0.06}Sb_{0.85}Te_3$ measured at different temperatures (2 K and 100 K) with an external magnetic field applied perpendicular the surface.

9 The temperature dependence of the magnetic susceptibility for $\mathbf{Bi_{1.09}V_{0.06}Sb_{0.85}Te_3}$

The temperature dependence of the magnetic susceptibility (χ), measured under an applied out-of-plane magnetic field of 1T in the range of temperatures from 2 K to 300 K with approximation by the Curie-Weiss law ($\chi = C/(T - \Theta)$) is presented in Fig.9S. The corresponding temperature dependence of $1/(\chi)$ with its linear approximation in the temperatures range below 100 K, shown in inset, clearly demonstrates a negative Weiss temperature (-4.2 K). It also testifies to the bulk AFM coupling at low temperatures.

The gradual decrease of the magnetic susceptibility with temperature and the developed AFM coupling in $Bi_{1.09}Gd_{0.06}Sb_{0.85}Te_3$ can be related to the contribution of the V d – Te p hybridization in formation of the magnetic properties. In Ref.[2] it was shown that the magnetic moment at the impurity ion induces a spin polarization on the neighboring Te and Sb host atoms. The resulting strength and kind of magnetic coupling (including AFM) depend on type of magnetic impurity, its concentration and stoichiometry of the TI.

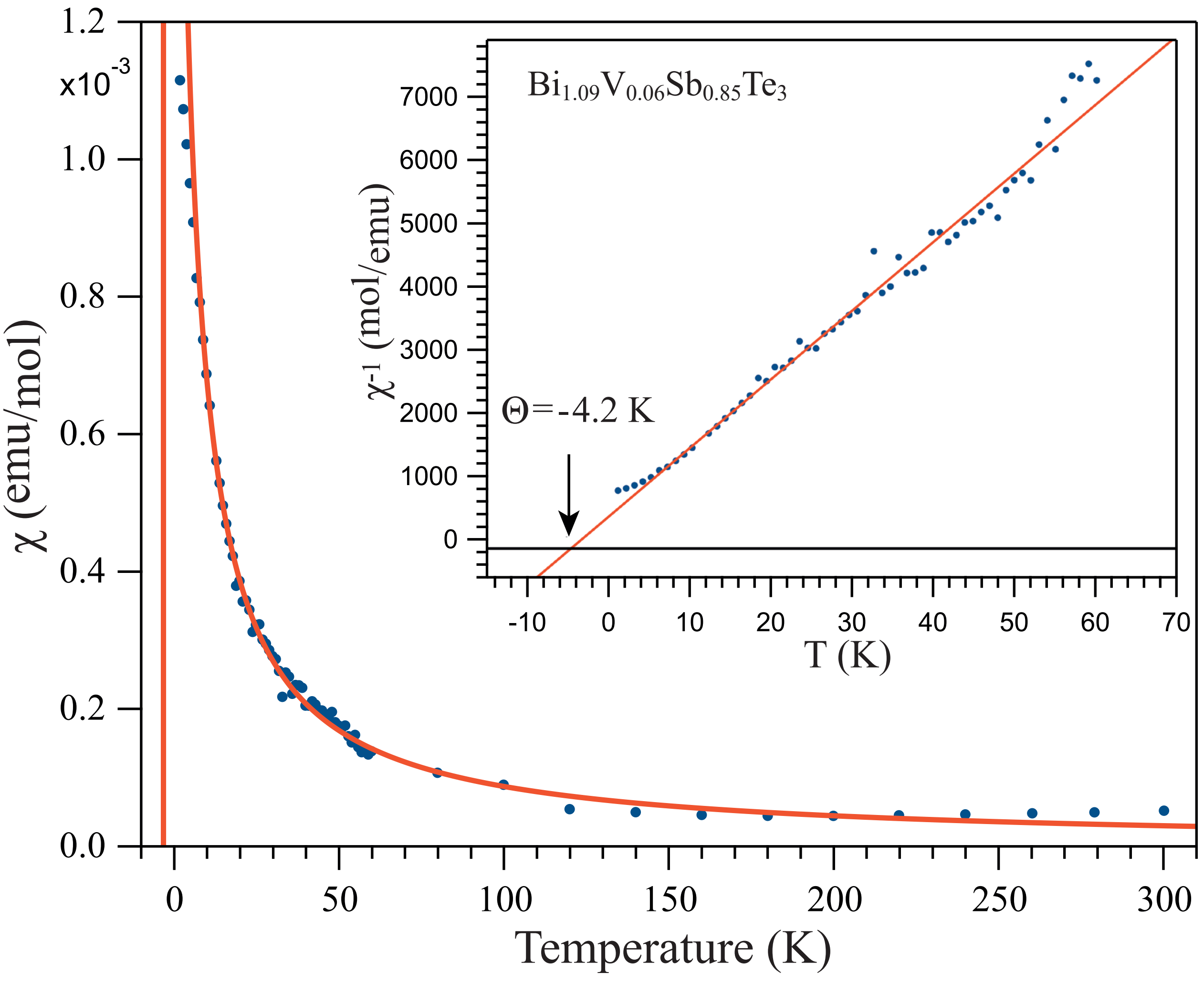

Figure 9. S The temperature dependence of the magnetic susceptibility (χ) measured for $Bi_{1.09}Gd_{0.06}Sb_{0.85}Te_3$ with an external magnetic field applied perpendicular the surface with approximation by the Curie-Weiss law at low temperatures (red lines). Inset - the temperature dependence of the inversed magnetic susceptibility $1/(\chi)$ with corresponding approximation at low temperatures (red lines) showing a negative values of the Weiss temperature ($\Theta = -4.2$ K).

10 $k_{\|}$-shift between the DC states measured under photoexcitation by circularly polarized SR of opposite helicity

Fig.10S shows the MDC profiles at different E_B measured for $Bi_{1.09}V_{0.06}Sb_{0.85}Te_3$ under photoexcitation by SR of opposite helicity which demonstrate the $k_{\|}$-shift between the DC states in relation to the accompanied upper DC state intensity redistribution.

The corresponding APRES dispersion maps are shown in insets. The cutting energies for measurements of the presented MDC-profiles are marked in the insets by horizontal lines. Comparison between the MDC profiles measured at opposite circular polarizations of SR shows an asymmetry in the DC states intensity at the DP inverted for opposite helicity of SR. It is followed by the corresponding $k_{\|}$-shift between the DC state maxima measured at opposite circular polarizations. The schematic presentations of the relation between the asymmetry in the DC state intensity asymmetry under photoexcitation (mappings at the Fermi level), the sign of the $k_{\|}$-shift of the DC states, and the induced in-plane magnetic field are shown in insets, too, for comparison. The value of the $k_{\|}$-shift at the DP is of about 3.5×10^{-3} Å^{-1}. (Unfortunately, for $Bi_{1.09}V_{0.06}Sb_{0.85}Te_3$ the features in MDC related to the opposite DC branches and the corresponding $k_{\|}$-shifts at other cutting energies outside the DP cannot be distinguished due to depletion of spectral weight of DC states with opposite momentum). However, a comparison at the DP demonstrates that the $k_{\|}$-shift for $Bi_{1.09}V_{0.06}Sb_{0.85}Te_3$ is almost two times lower than that for $Bi_{1.09}Gd_{0.06}Sb_{0.85}Te_3$ (5.8×10^{-3} Å^{-1}). It may be due to a smaller rotation of the magnetic moment for $Bi_{1.09}V_{0.06}Sb_{0.85}Te_3$ under photoexcitation by circularly polarized SR.

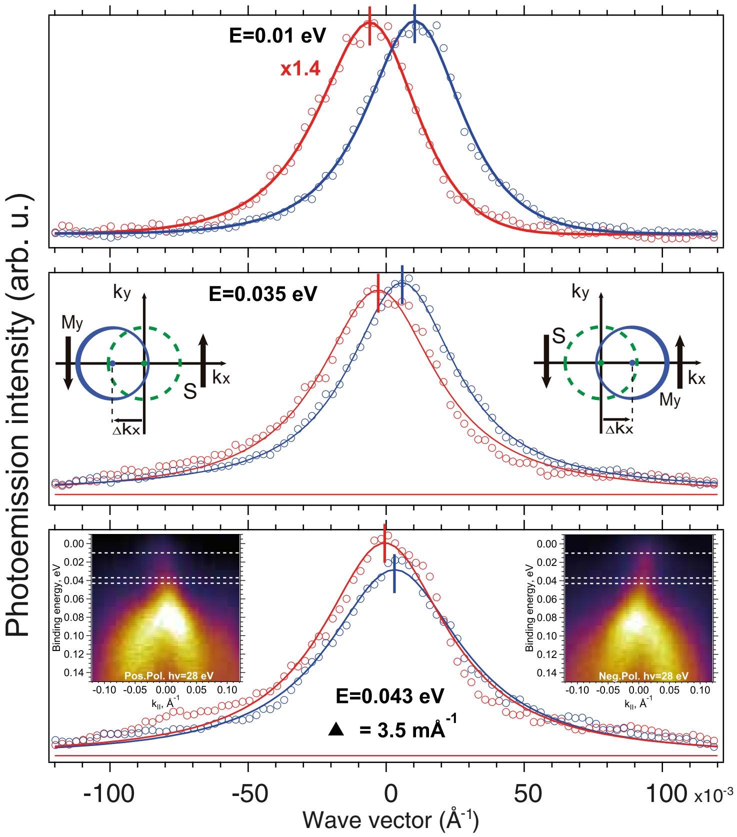

Figure 10. S MDC profiles of the DC states measured for $Bi_{1.09}V_{0.06}Sb_{0.85}Te_3$ at the DP and different cutting energies relative to the E_F under photoexcitation by circularly polarized SR of opposite helicity. The corresponding ARPES dispersion maps with the positions of the cutting energies marked by horizontal lines are shown in the insets at the bottom. The schematic presentations of the relation between the asymmetry in the DC state intensity under photoexcitation (mappings at the E_F), the sign of the $k_{\|}$-shift of the DC states and the induced in-plane magnetic field are shown in insets above, for comparison.

11 Estimation of the influence of the finite analyzer slit acceptance and corresponding line shape smoothing on the error in determining the DC state gap size

To determine the error introduced by the finite analyzer slit size on the correctness of the estimation of the gap at the DP using the experimental ARPES dispersion maps, we simulated the ARPES dispersion maps for different gaps taking into account the line shape smoothing and finite angle acceptance of the analyzer slit. The results of this simulation are presented in Supplementary Fig.11S. The modeling ARPES dispersion maps are shown in the forms of N(E) and d^2N/dE^2 in insets (1)-(3). The simulation was carried out for the Scienta analyzer slit size of 25×0.2 mm used in the current experiment (that corresponds to $\pm 0.07\circ$ of the angle acceptance or 3×10^{-3} Å^{-1}) for different values of the introduced gaps (0, 20 and 25 meV). Inset 4 shows the simulation of the ARPES dispersion maps for the slit size of 25×0.3 mm (that corresponds to $\pm 0.1\circ$ and the introduced gap of 20 meV. The modeling ARPES dispersion maps were calculated for the kinetic energy of 30 eV taking into account the spectral line width at half height of 45 meV that corresponds to the experimental broadening of the spectral lines. The simulation shows the results of smoothing over all angles (and corresponding spectra) which can be caught by analyzer with the slit noted above.

Below the ARPES dispersion maps the modification of the resulting EDC profiles at the DP (at $k_{\|} = 0$) are presented. One can see that the finite analyzer slit size in combination with the broadening of the experimental lines can lead to possible "observation" of artificial gap formation at the DP up to the value of 17 meV when the "real" gap is absent.

The introduction of the "real" gap at the DP of 20 and 25 meV in the modelling spectra is followed by the resulting increase of the artificial gap value up to 24 and 27 meV, respectively. In the case of a wider slit analyzer 25×0.3 mm and a real gap of 20 meV the artificial gap value increases not so much, up to 26 meV. It means that the gap less than 15 – 20 meV cannot be correctly measured by ARPES measurements at the used experimental condition. At the same time, when a "real" gap is open the resulting "measured" gap value increases on several meV.

As the real gap increases, the level of error in the gap value estimation by ARPES decreases. It means that the gap values of about 28 meV and 45 meV which can be distinguished from the experimental ARPES dispersion maps for $Bi_{1.09}Gd_{0.06}Sb_{0.85}Te_3$ and $Bi_{1.09}V_{0.06}Sb_{0.85}Te_3$, respectively, and its opposite changes up to 22 meV and 60 meV under photoexcitation by circularly polarized SR are associated with the real gap changes, while with possible error of about several meV. In the case when the Dirac gap is located near the E_F the error in estimating the gap value decreases (up two times) due to the absence or significantly reduced contribution from the states of the upper DC. It means that the experimental splitting between the DC states at the DP observed in the current work is certainly higher than the error caused by the finite size of the analyzer slit.

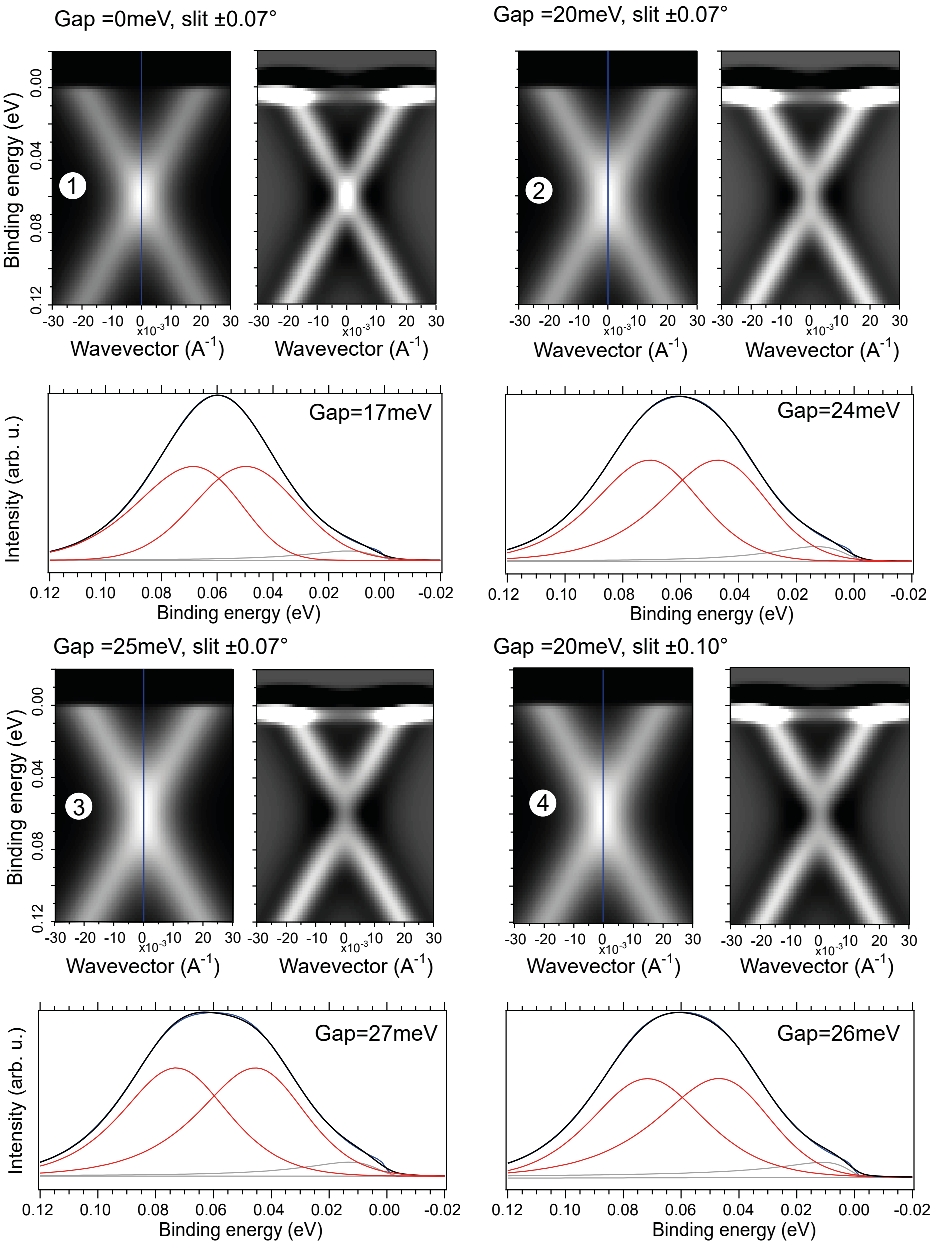

Figure 11. S Simulation of the ARPES dispersion maps in the region of the DP and dependence of “visible’ gap on the combinations of different introduced “real” gap value and the smoothing factor determined by the finite analyzer slit size. Below the ARPES dispersion maps in the $N(E)$ and d^2N/dE^2 forms the corresponding variation of the resulting EDC profiles are presented with decomposition into possible spectral components.

12 Estimation of the band gap at the Dirac point

To estimate size of the band gap at the Dirac point we use deconvolution for several peaks (2 or 3) of number of EDCs which were cut in vicinity of Γ-point. The same procedure of analysis and thus the same presentation were used for Fig.3,4,11 and Supplementary Fig.3S. Since the TIs are just slightly n-doped little part of the upper cone is visible. Therefore, peak from upper cone almost disappear at EDC at ± 0.01 Å^{-1} (± 0.02 Å^{-1} for $Bi_{1.09}Gd_{0.06}Sb_{0.85}Te_3$ in Fig.3). The EDCs at the 'maximum' distance from Γ-point in k-space with EDC exactly at the Γ-point are presented in b part of all Figs. In the part c dependence of split size between peaks form upper and bottom cone on wave vector is shown. We applied continuous fitting procedure to get parameters of the peaks near the G-point where they are hardly distinguished.

With intention to determine size of error and to achieve more proper size of the band gap the split dependence was fitted with a model function. Since the TSSs have the shape of cone in vicinity of the *Gamma*-point for approximation we used function like $\Delta = \mathrm{A} \cdot \sqrt{((k-k_0)^2 + \lambda^2)}$, where A is scale coefficient, k_0 is shift from 0 and λ is value proportional to the band gap size at $k = k_0$. From this estimation the band gap size is $\mathrm{Gap} = \mathrm{A} \cdot \lambda$. As error value we took square deviation of experimental spots from fitted function. The fitted function is shown by black dashed line in c part. Also it was noted that parameter A is about 4, if the upper cone disperses with k and about 2, if the upper cone is visible just as a spot. This leads to flatter split vs wave vector dependence (see Supplementary Fig.3S).

References

1. Vergniory, M. G. *et al.* Exchange interaction and its tuning in magnetic binary chalcogenides. *Phys. Rev. B* **89**, 165202, DOI: 10.1103/PhysRevB.89.165202 (2014).
2. Peixoto, T. R. F. *et al.* Impurity states in the magnetic topological insulator $V:(Bi,Sb)_2te_3$. *PRB* **94**, 195140 (2016).
